# Cross-reactive EBNA1 immunity targets alpha-crystallin B and is associated with multiple sclerosis

**DOI:** 10.1126/sciadv.adg3032

**Published:** 2023-05-17

**Authors:** Olivia G. Thomas, Mattias Bronge, Katarina Tengvall, Birce Akpinar, Ola B. Nilsson, Erik Holmgren, Tara Hessa, Guro Gafvelin, Mohsen Khademi, Lars Alfredsson, Roland Martin, André Ortlieb Guerreiro-Cacais, Hans Grönlund, Tomas Olsson, Ingrid Kockum

**Affiliations:** ^1^ Therapeutic Immune Design, Center for Molecular Medicine, Department of Clinical Neuroscience, Karolinska Institute, 171 76 Stockholm, Sweden.; ^2^ Neuroimmunology Unit, Department of Clinical Neuroscience, Center for Molecular Medicine, Karolinska Institute, 171 76 Stockholm, Sweden.; ^3^ Science for Life Laboratory, Department of Medical Biochemistry and Microbiology, Uppsala University, 75123 Uppsala, Sweden.; ^4^ Institute of Environmental Medicine, Karolinska Institute, 171 77 Stockholm, Sweden.; ^5^ Institute of Experimental Immunology, University of Zurich, Winterthurerstrasse 190, 8057 Zurich, Switzerland.

## Abstract

Multiple sclerosis (MS) is an inflammatory disease of the central nervous system, for which Epstein-Barr virus (EBV) infection is a likely prerequisite. Due to the homology between Epstein-Barr nuclear antigen 1 (EBNA1) and alpha-crystallin B (CRYAB), we examined antibody reactivity to EBNA1 and CRYAB peptide libraries in 713 persons with MS (pwMS) and 722 matched controls (Con). Antibody response to CRYAB amino acids 7 to 16 was associated with MS (OR = 2.0), and combination of high EBNA1 responses with CRYAB positivity markedly increased disease risk (OR = 9.0). Blocking experiments revealed antibody cross-reactivity between the homologous EBNA1 and CRYAB epitopes. Evidence for T cell cross-reactivity was obtained in mice between EBNA1 and CRYAB, and increased CRYAB and EBNA1 CD4^+^ T cell responses were detected in natalizumab-treated pwMS. This study provides evidence for antibody cross-reactivity between EBNA1 and CRYAB and points to a similar cross-reactivity in T cells, further demonstrating the role of EBV adaptive immune responses in MS development.

## Teaser

Multiple sclerosis-associated autoantibodies and T cells cross-react with EBNA1 and the CNS autoantigen alpha-crystallin B.

## INTRODUCTION

Multiple sclerosis (MS) is a chronic inflammatory autoimmune disease of the central nervous system (CNS), characterized by the migration of primarily adaptive immune cells across the blood-brain barrier and subsequent focal inflammatory demyelinating lesions (*1*). The etiology of MS remains incompletely understood, but it is believed to be caused by an interplay of environmental factors, for example, low vitamin D and smoking, and genetic risk factors where HLA-DRB1*15:01 and HLA-A*02:01 represent the strongest risk and protective genes, respectively (*2*, *3*).

It is well established that Epstein-Barr virus (EBV) infection is associated with an increased risk of MS and is a prerequisite for disease development (*4*–*6*). In addition, aspects of EBV infection interact with both genetic and environmental risk factors, and combinations are associated with markedly increased MS risk (*7*–*11*). The exact mechanisms of these associations are not fully understood, and as more than 90% of the general population is infected with EBV, but only a few develop MS, other underlying mechanisms must be at play (*4*). Fitting with observations that MS susceptibility genes primarily affect antigen presentation and effector T cell functions such as activation and growth (*12*), studies have shown differences in the host immune response to EBV infection in persons with MS (pwMS) compared to the population at large (*13*, *14*). One mechanistic explanation is molecular mimicry, where EBV-induced immune responses cross-target CNS proteins that contain epitopes with a similar amino acid sequence.

We previously found that antibodies against particular regions of EBV nuclear antigen 1 (EBNA1) were highly associated with MS, with odds ratios (ORs) of approximately four as opposed to two in other parts of the protein (*7*, *15*). One of the two highly associated fragments contained a peptide homolog to the autoantigen anoctamin 2 (ANO2) (*16*), with evidence of cross-reactivity as previously reported (*17*). The other fragment contained a sequence homology to the recently reported autoantigen glial cell adhesion molecule (GlialCAM) (*18*) and to the heat shock protein alpha-crystallin B (CRYAB) chain (*19*), leading to the current study that examined antibody cross-reactivity and T cell responses to this region.

CRYAB is expressed by oligodendrocytes in MS lesions and may have a protective effect by down-regulating proinflammatory responses of innate immune cells (*20*). Paradoxically, it has also been shown to be a target for adaptive immunity, and CRYAB’s protective effect on innate immunity could be reversed in the presence of a proinflammatory cytokine milieu (*20*–*23*).

Here, we examine anti-CRYAB immune reactivity in a large cohort of pwMS and controls (Con), map antibody epitopes, and explore possible cross-reactivity to EBNA1. Furthermore, we investigate CRYAB autoimmunity on the T cell level, both in an animal model and via a method previously used to detect autoreactive T cells in MS (*24*).

## RESULTS

### CRYAB autoantibodies are increased in MS

To explore anti-CRYAB autoantibodies, we used a suspension bead array to analyze circulating autoantibodies in plasma in a cohort of pwMS (*n* = 713) and population-based Con (*n* = 722) against stepped 15-mer peptides with a 14–amino acid overlap, covering the N terminus of CRYAB, four protein representations [three protein epitope signature tags (PrESTs) (*25*) and one full-length protein] (table S1). In addition, we included peptides and proteins of EBNA1 and ANO2, which we have previously reported with increased reactivity in MS (*17*).

**Fig. 1.**
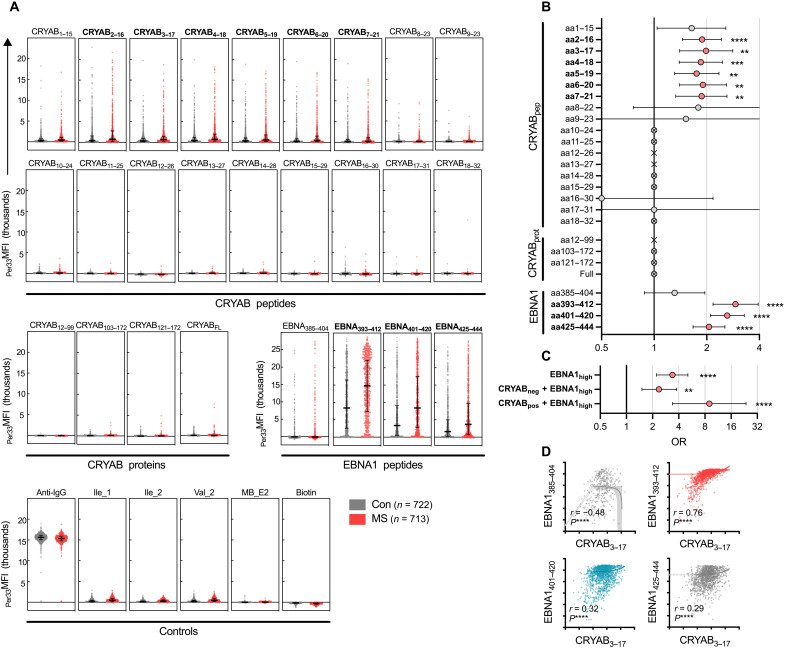
Increased anti-CRYAB IgG in MS. Suspension bead array measuring immunoglobulin G (IgG) against alpha-crystallin B (CRYAB) and Epstein-Barr nuclear antigen 1 (EBNA1) using plasma from a cohort of persons with multiple sclerosis (pwMS) (*n* = 713) and controls (Con) (*n* = 722). (**A**) Background-adjusted mean fluorescent intensity [33rd percentile MFI (_Per33_MFI)] values of CRYAB-stepped peptides (top panel), CRYAB protein fragments and CRYAB full length protein (CRYAB_FL_) (middle left panel), EBNA1 peptides associated with MS risk (middle right panel), and controls (bottom panel). Each dot represents one individual, and staples denote the median and interquartile range (IQR). (**B**) Odds ratio (ORs) of MS versus Con for the different reactivities in (A), with positive responses defined as >99.9th percentile of negative control peptide responses. ORs were calculated using the Baptista-Pike method with Fisher’s exact test for *P* values and Holm-Sidak correction for multiple comparisons. Staples denote 95% confidence interval (CI). ORs based on 1 or 0 events are depicted as crossed circles (>infinity) or as a cross (no OR). aa, amino acids. (**C**) ORs for combinations of antibody responses: EBNA1-high responders (defined as >median + 2 SD of Con response), EBNA1-high and CRYAB-negative responders, and EBNA1-high and CRYAB-positive responders. CRYAB positivity is defined as in (B) and is based on CRYAB_3–17_. The ORs were calculated as MS versus Con. For both (B) and (C), the exact number of positive and negative individuals is presented in table S2. (**D**) Correlation between CRYAB_3–17_ responses and EBNA1 responses (log_10 Per33_MFI). Spearman correlation coefficient (*r*) and *P* values are indicated. The lines and highlighted areas represent linear regression slopes and the 95% CI of slopes. For the whole figure, *P < 0.05; **P < 0.01; ***P < 0.001; ****P < 0.0001 (adjusted *P* values).

We detected increased anti-CRYAB immunoglobulin G (IgG) antibodies in pwMS targeting the peptides CRYAB_2–16_ up to CRYAB_7–21_, with similar trends seen for the adjacent CRYAB_1–15_, CRYAB_8–22_, and CRYAB_9–23_ peptides (Fig. 1, A and B). Setting a threshold for positivity at the 99.9th percentile of the negative control responses in the assay resulted in the highest OR for MS of CRYAB_3–17_ reactivity [OR: 1.98, 95% confidence interval (CI): 1.40 to 2.82, *P* = 0.0041] with 13.3% positive pwMS and 7.2% positive Con, respectively (Fig. 1B and table S2). CRYAB_2–16_ had the highest proportion of positive responses, with 27.6% positive pwMS and 16.9% positive Con [OR: 1.88, 95% CI: 1.45 to 2.43, *P* = 3.6 × 10^−5^). In contrast, very few positive signals were seen for other CRYAB peptides and proteins, which did not fully cover amino acids 9 to 15. Similarly, responses to full-length CRYAB were much weaker, possibly indicating a secondary protein structure that prevents antibodies from contacting a linear epitope.

The anti-CRYAB reactivity was similar in persons with relapsing-remitting, secondary progressive, and primary progressive MS and was not correlated with age, disease duration, or sex (fig. S1). Responses to CRYAB_3–17_ and EBNA1_292–412_ were increased in HLA-DRB1*15:01^+^ donors compared to negative individuals, indicating that these responses may be associated with this MS risk allele (fig. S1E). In addition, stratification of patients by self-reported history of infectious mononucleosis (IM) showed no difference in antibody reactivity to CRYAB_3–17_, EBNA1_393–412_, or EBNA1_401–420_ (fig. S1E).

On the basis of discrete, MS-associated significant responses to overlapping peptides between CRYAB_2–16_ and CRYAB_7–21_, we could identify a minimal MS-associated epitope of 10 amino acids with the sequence HPWIRRPFFP, which corresponded to CRYAB amino acids 7 to 16. Responses to these six peptides were highly correlated, pointing toward a single, distinct binding epitope (fig. S2). Because similar (but nonsignificant) trends were observed for the peptides CRYAB_1–15_, CRYAB_8–22_, and CRYAB_9–23_, a core epitope of only seven amino acids (CRYAB amino acids 9 to 15, WIRRPFF) may be able to bind antibodies but with a lower affinity.

### CRYAB and EBNA1 antibody responses interact and increase MS risk

As previously reported (*7*), anti-EBNA1 antibodies were also associated with MS, particularly anti-EBNA1_393–412_ (OR: 2.92, 95% CI: 2.17 to 3.94, adjusted *P* < 1 × 10^−14^), which includes the known sequence homology to both GlialCAM (*18*) and CRYAB. EBNA1_385–404_ and EBNA1_401–420_, covering only GlialCAM and CRYAB respectively, displayed lower ORs for MS compared to EBNA1_393–412_, as did the ANO2 homologous EBNA1_425–444_ fragment (Fig. 1B; homologies are depicted in fig. S3). EBNA1_393–412_ high response (defined as above median + 2 SD of Con responses) showed a greater association with MS (OR: 3.39, 95% CI: 2.22 to 5.10, *P* = 5.3 × 10^−8^) (Fig. 1C and fig. S4). For individuals with both CRYAB positivity (based on CRYAB_3–17_) and EBNA1_393–412_ high response, the OR for MS increased markedly (OR: 8.99, 95%CI: 3.39 to 23.76, *P* = 0.0052). Antibody reactivity to ANO2 was also analyzed and results were concurrent with our previous results (ANO2_134–153_, OR: 2.12, 95% CI: 1.63 to 2.77; fig. S4) (*17*).

All individuals with CRYAB_3–17_ reactivity were also positive for reactivity against EBNA1_393–412_, which was not the case for adjacent EBNA1 fragments (Fig. 1D). The opposite was true for ANO2_134–153_, which strongly correlated with EBNA1_425–444_ (the region to which it contains sequence homology) but not with the nonhomologous fragment EBNA1_393–412_ (figs. S2 and S4B).

### CRYAB autoantibodies cross-react with an homologous EBNA1 epitope

**Fig. 2.**
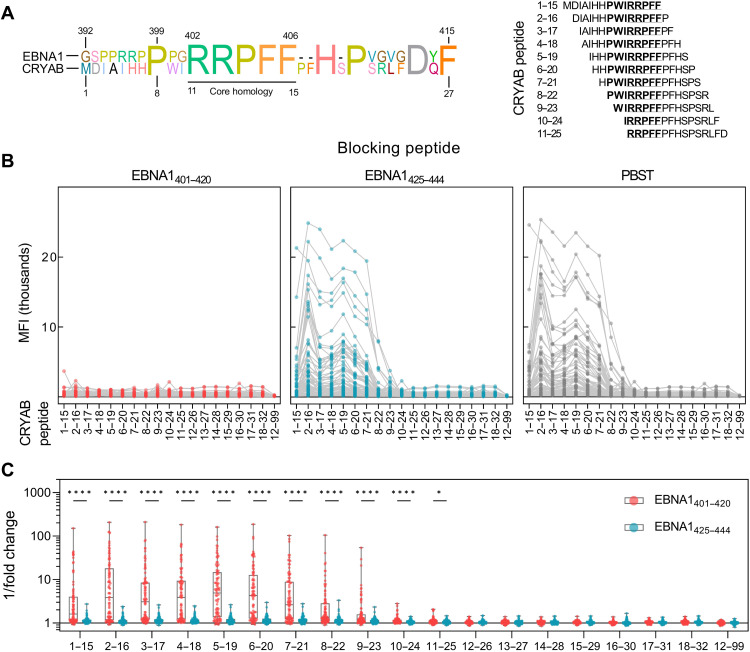
Anti-CRYAB antibodies cross-react with the homologous sequence in EBNA1. (**A**) Alignment of EBNA1_392–415_ and CRYAB_1–27_ amino acid sequences with the core homology between EBNA1_402–406_ and CRYAB_11–15_ underlined (left panel). Sequences of CRYAB peptides tested with homology are indicated in bold, and core sequences are underlined (right panel). (**B**) Anti-CRYAB IgG reactivity in pwMS (*n* = 91) after spiking plasma with EBNA1_401–420_, EBNA1_425–444_, or phosphate-buffered saline (PBS)–Tween 20 (PBST). Results are plotted as raw MFI. (**C**) Fold change of individual antibody responses after blocking with EBNA1_401–420_ or EBNA1_425–444_, compared to the PBST assay control [based on the data in (B)]. Presented as 1/fold change, i.e., higher values represent more efficient blocking. *P* values were calculated using multiple Wilcoxon signed-rank tests with Holm-Sidak correction for multiple comparisons. *P < 0.05; ****P < 10^−9^.

There is a high sequence homology between CRYAB amino acids 8 to 20 and EBNA1 amino acids 399 to 408, which have 8 of 13 identical amino acids counting gaps (alignment shown in Fig. 2A). In particular, the motifs in CRYAB amino acids 11 to 15 and EBNA1 amino acids 402 to 406 contain a core, homologous sequence “RRPFF” with five of five identical amino acids. Hence, cross-reactivity between EBNA1 and CRYAB fits with our data, as the core homologous region is within the binding epitope of CRYAB amino acids 7 to 16 defined by the epitope mapping.

To explore this further, we repeated the epitope mapping of CRYAB using plasma from pwMS (*n* = 91) that had been depleted of potential cross-reactive antibodies by spiking in the EBNA1 peptide, which contains the core homology to CRYAB (EBNA1_401–420_). EBNA1_425–444_, which shares homology with ANO2 but not with CRYAB, was spiked in as a control.

Spike in of EBNA1_401–420_ completely blocked reactivity to all CRYAB peptides and reduced responses to assay background levels (Fig. 2B). The reduction after addition of EBNA1_401–420_ was significant for all peptides containing the core homology when compared with EBNA1_425–444_ spike in (Fig. 2C), demonstrating that antibodies targeting EBNA1_401–420_ also bind CRYAB peptides containing the homologous motif. While EBNA1_401–420_ significantly blocked responses to all peptides containing the core homology amino acids 11 to 15 (RRPFF), the signal strength dropped for all peptides after CRYAB_8–22_, indicating that the shared proline residue (position 8 of CRYAB and position 399 of EBNA1; Fig. 2A) is important for CRYAB antibody binding, despite not being necessary for binding to EBNA1.

### EBNA1 and CRYAB immunization cross-primes reactive T cells to the reciprocal antigen

Because autoreactive T cells play an essential role in MS immunopathogenesis (*1*, *26*), we continued to explore whether a similar cross-reactivity between EBNA1 and CRYAB was present in the T cell compartment using a mouse model. Here, we used CRYAB- or EBNA_380–641_-immunized mice and examined T cells from draining lymph nodes for their antigen reactivity using a bead-bound antigen method (*27*).

**Fig. 3.**
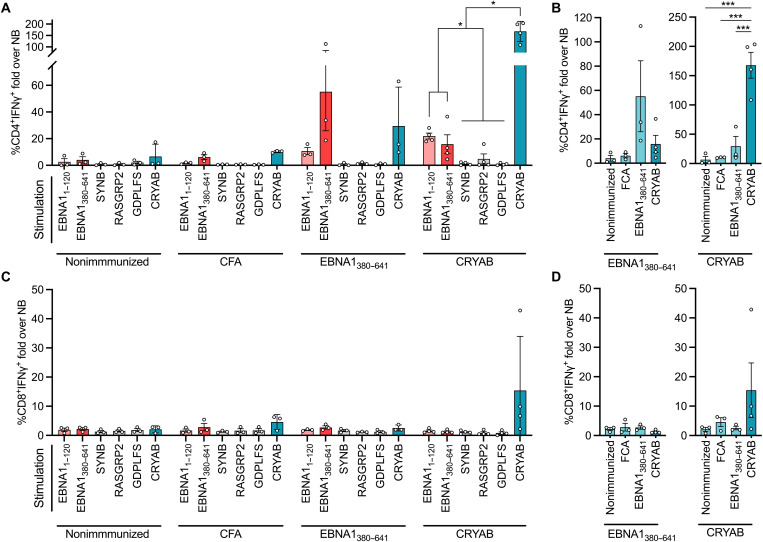
EBNA1 and CRYAB immunization cross-prime CD4^+^ T cells in vivo. Draining lymph node lymphocytes from nonimmunized mice (*n* = 3) or mice immunized with recombinant CRYAB (*n* = 4), EBNA1_380–641_ (*n* = 3), or Freund’s complete adjuvant (FCA; *n* = 3) were examined for antigen reactivity using recall stimulations and intracellular cytokine staining flow cytometry. (**A**) CD4^+^ T cells responding to restimulation after 10 days with bead-bound antigens. Data are presented as the fold change of %CD3^+^CD4^+^IFNγ^+^ over the naked bead (NB)–negative stimulation control. (**B**) Comparison of CD4^+^ responses within the EBNA1_380–641_ and CRYAB immunization groups. Data are presented as in (A). (**C**) Responses of CD8^+^ T cells from draining lymph node lymphocytes responding to restimulation as in (A). Data are presented as the fold change of %CD3^+^CD8^+^IFNγ^+^ over the NB negative control. (**D**) Comparison of CD8^+^ responses within the EBNA1_380–641_ and CRYAB immunization groups [as in (C)]. Each dot represents one biological replicate. *P* values were calculated using a two-way analysis of variance (ANOVA) with Tukey’s multiple comparisons test, comparing each different restimulation within the immunization group and indicated where significant. Bars and staples denote means ± SEM. *P < 0.05; ***P < 0.001. RASGRP2, RAS guanyl-releasing protein 2 (*29*); SYNB, synuclein beta (*28*); GDPLFS, GDP-l-fucose synthase.

EBNA1_380–641_-primed CD4^+^ T cells responded with interferon γ (IFNγ) production after stimulation with EBNA1_380–641_ and, to a lesser degree, EBNA1_1–120_. Of note, CD4^+^ T cells from EBNA1_380–641_-immunized mice also responded with increased IFNγ after stimulation with full-length CRYAB (Fig. 3 and repeated in fig. S5), while no response was seen after stimulation with either cytomegalovirus (CMV) pp65 or other MS-associated autoantigens [synaptosomal-associated protein 91 (*24*), synuclein beta (*28*), RAS guanyl-releasing protein 2 (RASGRP2) (*29*), and GDP-l-fucose synthase (GDPLFS) (*30*)]. Similarly, CD4^+^ T cells from CRYAB-immunized mice reacted to EBNA1_380–641_ and EBNA1_1–120_ but not to other MS-related autoantigens (Fig. 3, A and B). In contrast, neither unimmunized nor Freund’s complete adjuvant (FCA)–immunized mice responded to any of the antigens tested. However, CD8^+^ T cells from CRYAB- or EBNA_380–641_-immunized mice did not show significant reactivity to autoantigens compared to nonimmunized or FCA-immunized mice (Fig. 3 and fig. S5B).

### Increased proinflammatory CRYAB- and EBNA1-specific CD4^+^ memory T cells in natalizumab-treated pwMS

**Fig. 4.**
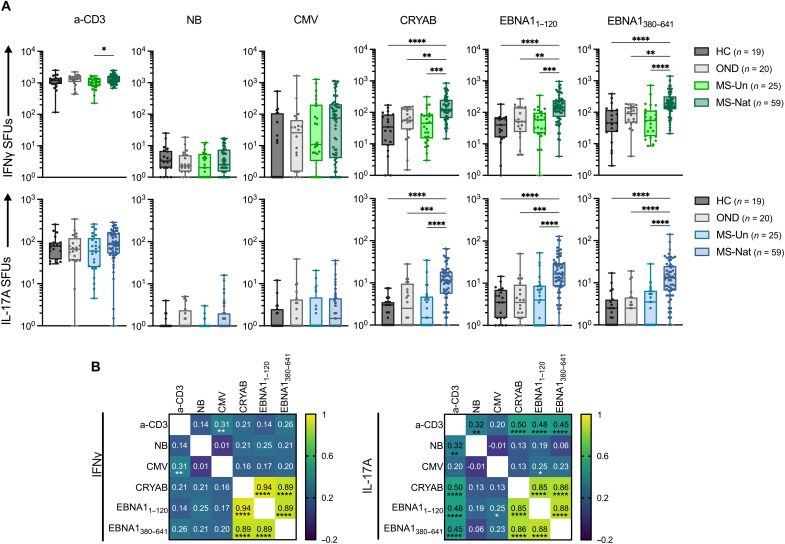
Increased circulating autoreactive CRYAB-specific cells in MS-Nat. (**A**) Number of interferon γ (IFNγ; top graphs) and interleukin-17A (IL-17A; bottom graphs) spot-forming units (SFUs) in a FluoroSpot assay after NB negative control, CRYAB, and EBNA1 stimulations. SFUs <1 are plotted as 1. Boxes represent median ± IQR. Statistical significance was calculated with a nonparametric, two-tailed Kruskal-Wallis test with Dunn’s multiple comparison test. Each group was compared with every other group, and *P* values are indicated where significant. *P < 0.05; **P < 0.01; ***P < 0.001; ****P < 0.0001. CMV, cytomegalovirus; MS-Un, untreated pwMS; MS-Nat, natalizumab-treated pwMS; HC, healthy control; OND, other neurological disease controls. (**B**) Correlation matrix of individual’s responses to the different stimulations [based on the data in (A)]. *r* and *P* values were calculated using nonparametric Spearman correlation with Holm-Sidak correction for multiple comparisons. Each comparison’s *r* value is written in the corresponding cell. *P* values are denoted when significant. *P < 0.05; **P < 0.01; ****P < 0.0001.

Next, we examined the frequency of CRYAB- and EBNA1-reactive T cells in natalizumab-treated pwMS (MS-Nat; *n* = 59), pwMS before starting disease-modifying treatment (MS-Un; *n* = 25), healthy control (HC; *n* = 19), and other neurological disease controls (OND; *n* = 20) using bead-bound antigen stimulations in an IFNγ/interleukin-17A (IL-17A)/interleukin-22 (IL-22) FluoroSpot assay, which we have previously used to detect autoreactive T cells (*24*). There was a notable increase in IFNγ^+^, IL-17A^+^, and IL-22^+^ cells responding to CRYAB, EBNA1_1–120_, and EBNA1_380–641_ in MS-Nat compared to both MS-Un and the control groups (Fig. 4A and fig. S6). In contrast, there were no differences in response to either CMV or naked beads (NB). A slightly higher polyclonal IFNγ response was seen in MS-Nat compared to MS-Un and IL-22 response in MS-Nat compared to OND but not compared to the other control groups. The response to both EBNA1 and CRYAB was similar in MS-Un and control groups.

Responses to EBNA1 and CRYAB were also highly correlated (Fig. 4B), while IFNγ control stimuli responses had little overlap. In contrast, IL-17A polyclonal responses also correlated with EBNA1 and CRYAB, reflecting the more heterogenous levels of IL-17A–producing cells (Fig. 4A, left-hand panels). Responses were similar regardless of sex, age, HLA-DRB1*15:01 status, disease duration, and expanded disability status score (fig. S7).

**Fig. 5.**
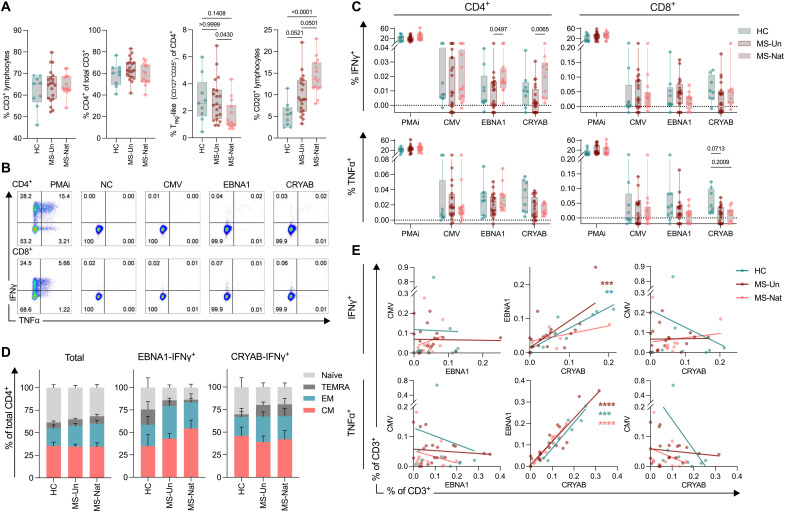
Characterization of CRYAB-responsive T cells. Spectral flow cytometry analysis of antigen-stimulated peripheral blood mononuclear cells (PBMCs) in a representative subcohort of the complete PBMC cohort presented in Fig. 4: HC (*n* = 9), MS-Un (*n* = 20), and MS-Nat (*n* = 14). (**A**) Bulk populations of lymphocyte subsets, based on NB-stimulated cells [negative control (NC) stimulation]. *P* values were calculated using a Kruskal-Wallis test with Dunn’s correction for multiple comparisons. T_reg_, regulatory T cell. (**B**) Representative plots for intracellular cytokine staining (ICS). (**C**) ICS of antigen bead-stimulated CD4^+^ and CD8^+^ T cells. All values presented are individually background adjusted (subtraction of NC-stimulated results). *P* values were calculated using a two-tailed Mann-Whitney *U* test with Holm-Sidak correction for multiple comparisons. PMAi, phorbol 12-myristate 13-acetate and ionomycin. (**D**) Distribution of central memory (CM; CCR7^+^CD45RA^−^), effector memory (EM; CCR7^−^CD45RA^−^), terminally differentiated EM (TEMRA; CCR7^−^CD45RA^+^), and naïve (CCR7^+^CD45RA^+^) in the total bulk CD4^+^ compartment and EBNA1- and CRYAB-responsive IFNγ^+^ CD4^+^ T cell compartments. Bars and staples represent the mean and SEM, respectively. Full data are presented in fig. S11. (**E**) Correlation of IFNγ and tumor necrosis factor α (TNFα) responses to CMV, EBNA1, and CRYAB. Lines and *P* values denote the linear regression curve. *P < 0.05; **P < 0.01; ***P < 0.001; ****P < 0.0001.

Using spectral flow cytometry, we further characterized CRYAB- and EBNA1-reactive T cells in a representative subcohort (gating strategy in fig. S8). While the bulk frequency of CD4^+^ T cells was similar in all groups, there were fewer regulatory T (T_reg_)–like cells (CD3^+^CD4^+^CD25^+^CD127^−^) and more B cells in MS-Nat, with similar trends in MS-Un (Fig. 5A). The increased B cells were primarily of memory phenotype (fig. S9). Increased frequencies of CXCR3^+^ B cells were also observed in MS-Nat, a phenomenon that has previously been reported in MS (fig. S9) (*31*).

Using intracellular cytokine staining, increased anti-CRYAB IFNγ^+^ CD4^+^ T cells were again detected in MS-Nat compared to MS-Un with similar but nonsignificant trends compared to HC (Fig. 5, B and C). In contrast, there were no differences in control stimulations or in the CD8^+^ compartment. This trend for increased CRYAB responses in MS-Nat individuals was specific to the CD4^+^IFNγ^+^ compartment, whereas responding populations with CD4^+^TNFα^+^, CD8^+^IFNγ^+^, or CD8^+^TNFα^+^ phenotypes were slightly increased in the HC group (Fig. 5C).

CRYAB- and EBNA1-responsive CD4^+^ T cells were primarily of effector memory (EM) and central memory (CM) phenotypes (Fig. 5D and figs. S10 and S13), indicative of being antigen experienced. Similar in the FluoroSpot assay, individual EBNA1 and CRYAB responses were highly correlated with each other but not with the CMV response (Fig. 5E). Trends of decreased tumor necrosis factor α (TNFα), a possibly protective cytokine in neuroinflammation (*32*), responses toward CRYAB were also seen in MS-Nat, suggesting a switch to a more pathological T cell phenotype in the CRYAB-responsive CD4^+^ compartment in pwMS (Fig. 5, C and E). Similarly, in MS-Nat and MS-Un groups, CRYAB-reactive CD4^+^IFNγ^+^ cells contained a higher proportion of memory phenotypes, especially the terminally differentiated EM (TEMRA) T cells, which was not the case in EBNA1- or CMV-responsive cells (Fig. 5E and fig. S10). Granulocyte-macrophage colony-stimulating factor and IL-17A responses were also analyzed with low responses and no apparent differences in this experimental setup (fig. S11). Together, these data demonstrate an increased EBNA1- and CRYAB-specific proinflammatory CD4^+^ memory T cell population in MS-Nat, with highly correlated responses indicative of cross-reactivity.

## DISCUSSION

There is a clear epidemiological link between MS and EBV infection, but the mechanistic understanding of this relationship remains incomplete (*6*). One plausible hypothesis is molecular mimicry between EBV antigens and CNS autoantigens, a previously demonstrated phenomenon at both the antibody and T cell level in a subgroup of pwMS (*17*, *18*, *33*–*35*). Here, we provide evidence of increased autoimmunity targeting the N-terminal end of CRYAB in MS with approximately 13.2 to 27.6% IgG seropositivity in pwMS and demonstrate that these autoantibodies cross-react with the homologous and MS-associated region of EBNA1 (amino acids 402 to 406) (*7*). Furthermore, high EBNA1 responses when combined with seropositivity for anti-CRYAB were associated with a markedly increased risk of MS with an OR of 9.0. As discussed below, we speculate that this increased association may depend on other peptide mimics of CNS antigens within EBNA1. We also provide evidence of increased and highly correlated T cell responses to CRYAB and EBNA1 in MS-Nat and show that EBNA1-primed T cells are sensitized to CRYAB, indicating additional cross-reactivity of cellular responses.

This study provides further evidence that molecular mimicry contributes to pathological mechanisms linking EBV with MS. EBNA1 has several different homologies in a narrow region. One study demonstrated cross-reactivity between EBNA1 amino acids 394 to 399 and GlialCAM (*18*), and we have previously demonstrated the same for EBNA1 amino acids 431 to 440 with ANO2 (*17*). In addition, EBNA1 amino acids 411 to 426 and myelin basic protein cross-reactivity has been demonstrated in experimental autoimmune encephalomyelitis (EAE) (*36*), and EBNA1-specific T cells have been shown to react to a mixed myelin antigen pool (*33*). We now demonstrate further cross-reactivity between EBNA1 amino acids 402 to 406 and CRYAB amino acids 11 to 15, with core sequence homology mapped to identical RRPFF residues within these fragments. While many proteins contain this core amino acid RRPFF motif, amino acid residues that flank this sequence and are unique to CRYAB are also necessary for antibody binding. This is evidenced by the drop in signal after CRYAB amino acids 8 to 22, indicating that the shared proline at CRYAB position 8 (and at EBNA1 position 399) is critical to the epitope.

Several different autoantigens may be involved in mimicry between MS and EBV, which could explain why each study to date has found autoreactivities in only ~20% of pwMS, despite essentially ubiquitous EBNA1 responses. In support of this, we saw a poor correlation between CRYAB and ANO2 IgG responses despite the shared EBNA1 link, which suggests that underlying factors such as human leukocyte antigen (HLA), previous antigen experience of the immune system, history of IM, or other perhaps undiscovered risk factors affect the different cross-reactivity patterns. One study found MS discordant twins to have increased serum reactivity to EBNA1 amino acids 401 to 411, which overlaps with the CRYAB homologous epitope (*37*), an epitope that has also been reported as transiently immunodominant during IM but lower in healthy long-term virus carriers (*38*). Therefore, persistence of antibody reactivity to this epitope following resolution of IM could imply a defect in the affinity maturation process. Our study did not show an effect of previous IM on levels of antibody reactivity to EBNA1 amino acids 393 to 412, an effect that is consistent with our previous observation that a history of IM can act independently from the risk associated to EBNA1 titers (*10*). Our data also show that there is no enrichment of antibody responses to EBNA1 amino acids 385 to 404, the epitope that only included the GlialCAM homologous peptide, in HLA-DRB1*15:01^+^ individuals in our cohort, indicating that responses are not associated with this allele. We also note that, while our study analyzed antibody responses in a Swedish cohort of pwMS and population-based, matched Con, we detected CRYAB antibody responses in a small proportion of control participants, and therefore further analysis of other neurological disease cohorts is needed to determine whether these antibody responses are MS specific.

We also note that there may also be additional changes besides the increase of autoantibodies, such as the presence of CRYAB-reactive T cells observed in MS-Nat in the present study. The generation of high-affinity antibodies depends on T cell help, and therefore autoantibodies that target intracellular antigens, such as those directed against ANO2, may not be directly pathogenic (*17*) but rather markers of a T cell response that is also able to recognize intracellular antigens with pathogenic consequences. Similarly, autoantibodies targeting intracellular autoantigens as markers for a destructive effector T cell responses are present in other autoimmune diseases such as diabetes mellitus (*39*) and Addison’s disease (*40*). It has also been reported that B cells expressing surface Ig with specificity for an antigen are able to process and present this antigen with increased efficiency to T cells, which respond to the same target (*41*). This implies that presence of autoantibody responses may also lead to priming of pathogenic autoreactive T cell responses and could potentially lead to epitope spreading between humoral and cellular adaptive arms of the immune system. The increased ORs associated with MS, which require studies in large cohorts such as this one, indicate a pathogenic role both for the currently explored CRYAB and previously reported ANO2-immune reactivities in MS and provides further evidence for molecular mimicry in disease pathogenesis.

The historical role of CRYAB as an autoantigen in MS has been controversial since its first discovery almost 30 years ago (*23*). While initially proposed as an important autoantigen in neuroinflammation (*21*), animal studies have demonstrated its protective and even therapeutic effects, which are not limited to autoimmune diseases, likely by binding proinflammatory proteins (*20*, *42*, *43*). In addition, one study demonstrated the ability of several heat shock proteins including CRYAB to bind antibodies in a specificity-independent manner, casting doubt on previous results regarding humoral responses (*44*). This was demonstrated using full-length CRYAB but even short peptides such as amino acids 73 to 92 have been shown to exhibit chaperone activity (*45*), and other fragments including amino acids 9 to 20 exhibit protein interactions (although this has not been confirmed for antibodies) (*46*). Instead, we detect responses to CRYAB_3–17_ in our assays that does not include the amino acids 73 to 92 region previously reported to have chaperone activity, although we recognize that this does not exclude the possibility of protein:protein interaction activity. However, a nonspecific antibody:CRYAB peptide interaction would not explain the cross-reactivity between the homologous EBNA1 and CRYAB peptides that we demonstrate in this study nor the heterogeneity of CRYAB responses or the association with MS. If this were the case, then nonspecific binding would be even more pronounced for the CRYAB protein and PrESTs, as they include more of the interactive sites; however, we observed essentially no detectable IgG responses to larger CRYAB fragments. Last, the strongest responses were detected to peptides with only partial overlap with the reported interactive site amino acids 9 to 20 (i.e., CRYAB_2–16_) compared to the peptides with complete overlap, and this finding replicates a previous study that detected intrathecal antibody reactivity to CRYAB_1–15_ (*19*). Together, it is possible that CRYAB has a neuroprotective function, which could explain its treatment effect in neuroinflammatory models, while autoreactivity against it could reverse this role as previously indicated by Bsibsi *et al*. (*22*). Thus, a role for CRYAB in both neuroprotection and also as an autoantigen target driving autoimmunity is not mutually exclusive.

We could not detect increased T cell responses to CRYAB in untreated pwMS; it was only observed in natalizumab-treated pwMS. We believe that this represents a true increase in CRYAB T cell responses, since there were no increased responses to control antigens in this cohort. Because natalizumab blocks VLA-4–dependent migration (*47*) necessary for trafficking into the CNS and also into the gut, it is possible that these cells are particularly migratory and are therefore not present in the peripheral blood of untreated pwMS. It is therefore conceivable that precursor frequencies of CRYAB-specific T cells are below the detection limit for our assay in untreated pwMS. Furthermore, the strongly increased autoproliferation in natalizumab-treated pwMS (*29*) that results from B and T cell interactions could be involved in expanding CRYAB-specific T cells to above the limit of detection. While increased CRYAB-specific IFNγ responses were seen in both FluoroSpot and flow cytometry analysis, we could not replicate T helper cell 17–related cytokines in the latter assay. Again, this is likely due to assay detection limits, as IL-17A responses were an order of magnitude rarer than IFNγ in the FluoroSpot, and flow cytometry is known to be less sensitive than FluoroSpot, particularly at differentiating low-level responses from background signals (*48*).

The animal model data provide additional information regarding the possible roles of CRYAB-specific T cells. Previous studies showed that CRYAB-specific T cells are not able to induce neuroinflammatory disease in mice (*49*). However, the study used full-length protein for mouse immunization, and specific epitopes could play a role in neuroinflammation. Along this line, one study investigating T cells targeting the cryptic epitope CRYAB amino acids 1 to 16, which covers the cross-reactive region, observed mild encephalitogenic effects (*50*). In contrast, T cell epitopes in other regions of CRYAB remained nonencephalitogenic. Intermolecular epitope spreading from myelin basic protein to CRYAB and proinflammatory CD4^+^ responses have also been demonstrated in a spontaneous EAE model (*51*). However, the lack of encephalitogenicity in certain mouse strains does not exclude encephalitogenicity in others; for example, myelin oligodendrocyte glycoprotein is now the most established autoantigen in neuroinflammatory disease models, and different epitopes within this autoantigen are highly strain-dependent in their ability to induce EAE (*52*, *53*).

We have previously shown that CNS autoantigen-specific T cells remain at similarly high frequencies despite several years of natalizumab treatment, although they theoretically should not encounter their target antigens (*24*). This is in line with the high risk of relapse after treatment discontinuation (*54*) and suggests that disease-driving T cells are maintained in the peripheral immune system, which could also drive the high frequency of CRYAB-specific T cells observed in natalizumab-treated pw MS in the present study. Stimuli that maintain this population may come from recognition of cognate antigen or structurally similar autoantigens, possibly via EBV-infected B cells or via gut or lung microbiota, which is consistent with the hypothesis that molecular mimicry in the periphery primes CNS autoimmunity (*34*, *55*, *56*). Alternatively, or in addition, the recently described “self-activation” and subsequent autoproliferation of proinflammatory memory B cells and CD4^+^ T cells enriched for brain-homing cells may be involved (*29*). As CD20^+^ B cells constitute the main reservoir of EBV in chronic infection (*4*), part of the effectiveness of B cell depletion treatments may lie in removing this reservoir of antigens maintaining the autoreactive T cells in the periphery as shown by Jelcic *et al*. (*29*). One study showed that EBV induces increased CRYAB expression and HLA-DR presentation in infected B cells, supporting this hypothesis (*57*).

In conclusion, this study supports the role of CRYAB as an autoantigen in MS and demonstrates that CRYAB autoreactivity is cross-reactive with EBNA1 due to molecular mimicry, likely a result of EBV-targeted immunity. This provides further evidence regarding the mechanistic link between EBV infection and MS.

## MATERIALS AND METHODS

### Experimental design

The objective of this study was to investigate adaptive immune responses to CRYAB in MS and control participants and, as there is a sequence homology between CRYAB and the EBV antigen EBNA1, to determine whether responses to EBNA1 are also able to target CRYAB via molecular mimicry. We used a blinded suspension bead array to investigate antibody responses to both peptides and longer protein sequences from CRYAB and EBNA1 in both pwMS and population-based Con. Experimental results were correlated retrospectively with clinical data. Blocking experiments were used to determine the ability of CRYAB-specific autoantibodies to also bind homologous or nonhomologous peptides from EBNA1. The potential of T cells in the peripheral blood to respond to full-length CRYAB was verified using two different immunological methods alongside the EBNA1-specific T cell response, and their phenotype was characterized and correlated in individuals. Furthermore, evidence for T cell cross-reactivity was investigated in a mouse model, where EBNA1-immunized mice were then rechallenged with CRYAB and other previously described autoantigens to investigate T cell priming in vivo.

### Study participants

Plasma samples were obtained from the Swedish nationwide Epidemiological Investigation of MS cohort (*58*). In total, 713 pwMS and 722 Con were included. All participants provided written informed consent to sample collection and data analysis. Con were population-based controls matched to cases on sex, age, and geographic region. Cohort characteristics are available in table S3. HLA data were available for the cohort from previous studies at our institution, and IM history was self-reported in a questionnaire answered at the time of consent and sampling (*17*). For the T cell studies in MS, a cohort consisting of MS-Un (*n* = 26), or MS-Nat (*n* = 66), age- and sex-matched HC (*n* = 21), and persons with OND (*n* = 20, cases of narcolepsy type 1 or 2 and idiopathic hypersomnia) was collected. Some were excluded from analysis due to technical problems (see “FluoroSpot” section for further details), and cohort details (presented in table S3) reflect actual analyzed individuals (MS-Nat, *n* = 59; MS-Un, *n* = 25; HC, *n* = 19; and OND, *n* = 20). Peripheral blood mononuclear cells (PBMCs) were collected from venous blood samples using density gradient (Ficoll) separation and cryopreserved at −150°C until use in experimental assays. The study was approved by the Swedish Ethical Review Authority’s Stockholm Ethics Board (no. 04-252/1-4 and no. 2009/2107-3112) and the Cantonal Ethics Committee of Zürich (no. 2013-0001).

### Suspension bead array

To evaluate anti-CRYAB antibodies, a suspension bead array was used as previously described (*17*). Peptides were designed as 15 to 20 mers, conjugated to a biotin and amino hexanoic acid spacer (PEPscreen, Sigma-Aldrich). For CRYAB, overlapping 15-mer peptides which covered the N-terminal amino acids 1 to 32 with 14–amino acid overlap (total of 18 peptides) were used. In addition, PrESTs from The Human Protein Atlas library and a commercial full-length version was used (full list in table S1). For EBNA1, peptides covering both the N- and C-terminal part of EBNA1 (omitting the G-A repeat region amino acids 120 to 360), with a larger overlap for the MS-associated region amino acids 385 to 420, as well as previously reported EBNA1 cross-reactive ANO2 peptides were used. In addition, recombinant short protein fragments covering both cross-reactive and non−cross-reactive areas were included as well as longer protein representations (fig. S4 for all tested peptides and proteins).

Peptides and proteins were coupled to NeutrAvidin-coated color-coded magnetic beads (MagPlex, Luminex Corp.). As controls, biotin only (Sigma-Aldrich, B4501-5G), four peptides with low reactivity probability, 6xHis-ABP (purification tag for PrESTs and proteins), buffer only, and anti-human IgG (rabbit; Jackson ImmunoResearch, 309-005-082) were used. Plasma samples were diluted 1:150 in phosphate-buffered saline (PBS) supplemented with 5% bovine serum albumin, 0.1% Tween 20, 6xHis-ABD (160 μg/ml), and NeutrAvidin (10 μg/ml) to deplete potential tag-binding antibodies and were incubated for 1 hour followed by 2 hours of incubation with the bead array. Antibody binding was fixed with 0.2% formaldehyde for 10 min before the addition of a secondary antibody (anti-human IgG goat fab fragment conjugated to phycoerythrin ; H10104, Invitrogen) and 30 min of incubation. Samples were then read using a FLEXMAP 3D (Luminex Corp.).

Median bead mean fluorescence intensity (MFI) for each plasma sample was adjusted on the basis of the assumption that 33% of antigens would not give a positive signal, and the 33rd percentile MFI (_Per33_MFI) was subtracted from each response.

PwMS and Con samples were randomized on plates before the assay, and the assay was performed blinded regarding disease or control status. The whole cohort was performed on four different plates.

### EBNA1/CRYAB blocking

To explore the cross-reactive potential of anti-CRYAB antibodies, plasma from a subset of the suspension bead array cohort (pwMS, *n* = 91) was analyzed. The experiment was performed as described in the “Suspension bead array” section, with one modification. Before mixing plasma samples with the bead array, plasma samples were preincubated with the peptides EBNA1_401–420_ and EBNA1_425–444_ at 30 μM or an equivalent volume of PBS with 0.05% Tween 20 for 1 hour at room temperature.

### Production of recombinant antigens

For the FluoroSpot, full-length recombinant CRYAB, EBNA1_1–120_, and EBNA1_380–641_ (omitting the G-A repeat region for expression purposes) were produced as previously described (*27*). The gene covering the whole CRYAB (amino acids 1 to 175; UniProt identifier: P0251) including flanking Bsa I sites was ordered from Eurofins Scientific (Luxemburg) and subcloned into a modified pET28 vector containing an 8x histidine repeat for purification purposes in a one-step digestion-ligation reaction using the Bsa I sites and T4 DNA ligase as described (*59*). The vector was transformed into BL21-AI *Escherichia coli* (Thermo Fisher Scientific, cat. no.: C607003) and grown in a two-step culture. First, 4 ml of superbroth (SB) medium was inoculated with *E. coli* before being transferred to 50 ml of Vegitone SB supplemented with carbenicillin (100 mg/liter), 1 mM MgSO_4_, and 0.6% glycerol and incubated at 37°C for 3 hours. The culture was then transferred to 450 ml of SB supplemented with 1 mM MgSO_4_, 0.6% glycerol, carbenicillin (100 mg/liter), 0.015% glucose, 0.2% arabinose, and 0.2% lactose for autoinduction; and the cells were grown overnight at 20°C. The bacterial pellet was collected by centrifugation at 7000*g* for 30 min and frozen at −20°C.

Bacteria were lysed by thawing the pellets and addition of lysis buffer, followed by sonication for 5 min, and protein lysates were clarified by centrifugation for 1 hour at 20,000*g*. Supernatants were filtered through 0.45-μm filters before being loaded on 1 ml of Ni columns (ÄKTAxpress), washed, and eluted using an imidazole buffer followed by a second size exclusion purification step. SDS–polyacrylamide gel electrophoresis (SDS-PAGE) analysis of CRYAB showed high purity with dimerization (extra band at double the size of main band) (fig. S12A). An analysis of the amino acid sequence in SnapGene (Dotmatics) predicted two putative dimerization sites. EBNA1_1–120_ and EBNA1_380–641_ were produced similarly but were eluted from the Ni column using a pH 2 buffer and without the extra size exclusion step. Purity was similarly tested using SDS-PAGE (fig. S12A).

Antigen beads were prepared essentially as previously described (*27*, *60*). In short, the purified antigen was coupled to 1-μm paramagnetic beads (Dynabeads MyOne Carboxylic Acid, Thermo Fisher Scientific) using *N*-hydroxysuccinimide−1-ethyl-3-(3-dimethylaminopropyl)carbodiimide coupling, endotoxin was removed by a sodium hydroxide wash, and coupling efficiency was analyzed via flow cytometry on a Guava easyCyte (Luminex) after staining the 8x histidine tag of bead-coupled protein with nitrilotriacetic acid−Atto 488 (Merck/Sigma-Aldrich, no. 39625). Bead coupling efficiency was 73.5, 99.3, and 95.3% for CRYAB, EBNA1_1–120_, and EBNA1_380–641_, respectively (fig. S12B).

### FluoroSpot

To analyze the presence of CRYAB-specific T cells, an IFNγ/IL-22/IL-17A FluoroSpot was used. Cryopreserved PBMCs were briefly thawed in a 37°C water bath before washing twice in complete RPMI (cRPMI) [RPMI 1640 (Sigma-Aldrich, R8758) supplemented with 2 mM l-glutamine (Sigma-Aldrich, G7513) and penicillin (100 U/ml) and streptomycin (100 μg/ml; Sigma-Aldrich, P4333)]. Cell count and viability were analyzed using trypan blue staining in an automated cell counter (LUNA-II, Logos Biosystems) (fig. S12C). A total of 250,000 PBMCs in 200 μl of cRPMI were seeded per well in a precoated 96-well FluoroSpot plate (FSP-011803, Mabtech) before addition of CRYAB beads at a ratio of 10 beads per PBMC. Anti-CD3 antibody as a polyclonal stimulation, CMV beads (*27*) as an antigen-specific positive control, and NB as negative control were similarly added. For the anti-CD3, only 125,000 PBMCs were seeded per well. All conditions were performed in duplicates, and pwMS and HC were mixed on each plate to reduce potential plate effects. The plates were incubated for 44 hours at 37°C and 5% CO_2_ before development according to the manufacturer’s instruction. The developed plates were kept in the dark and allowed to dry before being read in an IRIS reader (Mabtech, Sweden). Each individual’s background response (NB) was subtracted from antigen responses before analysis and is presented as delta spot forming units (ΔSFUs). Because of insufficient amount of PBMCs, two HCs and four MS-Nat were excluded due to incomplete dataset. Three MS-Nat and one MS-Un were excluded from data analysis due to high background responses (>30 IFNγ spots in the NB-stimulation). The reported results and cohort characteristics reflect the analyzed cohort after exclusions.

### Spectral flow cytometry

To analyze the phenotype and cytokine production of antigen-specific T cells in peripheral blood, we used a 24-marker spectral flow cytometry panel. Cryopreserved PBMC samples were selected, and a representative subcohort consisting of HC (*n* = 8), MS-Nat (*n* = 14), and MS-Un (*n* = 20) were analyzed. PBMCs were prepared essentially as previously described for FluoroSpot. Briefly, 5 × 10^5^ PBMCs in 200 μl of cRPMI per well were seeded per well in a 96-well U-bottom plate, and stimuli were subsequently added. Instead of anti-CD3, a cocktail of phorbol 12-myristate 13-acetate and ionomycin (PMAi; Invitrogen, eBioscience, 00-4970-93) was used as a positive control. After 1 hour, brefeldin A (1 μg/ml; BioLegend, 420601) was added followed by incubation at 37°C and 5% CO_2_ for 16 hours. PBMCs were washed twice with 200 μl of PBS and stained for viability using ViaDye Red (Cytek Bioscience) for 20 min at 4°C, followed by one additional wash in PBS and one in magnetic-activated cell sorting (MACS) buffer (Miltenyi, 130-091-222), before resuspension in 25 μl of MACS buffer. Five microliters of Super Bright Complete Staining Buffer (eBioscience, SB-4401-75) was added per well for 5 min at room temperature followed by staining with a cocktail of surface marker antibodies (listed in table S4) for 30 min at 4°C. Following incubation, cells were washed once with 150 μl of MACS buffer and then once with 200 μl of MACS buffer. Fixation was then performed by resuspending PBMC in 200 μl per well of Cytofix buffer (BD Biosciences, 51-209KZ) before incubation at 4°C for 20 min. Following incubation, cells were washed twice with 200 μl of 1× Cytoperm buffer (BD Biosciences, 51-2091KZ) before resuspension in a cocktail of intracellular antibodies (table S4) and incubation for 30 min at 4°C. Cells were then washed twice with MACS buffer (first with 150 μl followed by 200 μl) and resuspended in 200 μl of MACS buffer before acquisition on a Cytek Aurora 4L instrument (Cytek Biosciences). SpectroFlo software (Cytek Biosciences) was used for correcting spectral overlap (unmixing), and data were analyzed in FlowJo v10 (BD Biosciences) and Cytobank (Beckman Coulter). All centrifugation steps before fixation were performed at 300*g* for 5 min, and following fixation, this was increased to 500*g*. Gating strategy is presented in fig. S8.

### Mouse immunization

Female SJL/J mice were purchased from Taconic Biosciences. All animals were housed in polystyrene cages containing aspen shavings, with ad libitum access to standard rodent chow and water. The cages were kept in temperature regulated rooms with a 12-hour light/dark cycle. The mice were immunized subcutaneously at the base of the tail with FCA (*n* = 8), 50 μg of EBNA1_380–641_ (*n* = 9), 50 μg of CRYAB (*n* = 4), or nothing (*n* = 8). Control and test mice were equally distributed among three separate experiments, apart from CRYAB-immunized mice that were only included in the final experiment. A standardized method to prepare adjuvant/antigen emulsions to induce autoimmune disease models has recently been published (*61*). Accordingly, to induce EAE, POWER-Kits were purchased from BTB Emulsions, Malmö, Sweden (https://btbemulsions.com/), and emulsions were prepared according to the manufacturer’s recommendations. The animals were euthanized after 10 days for antigen recall experiments. The study was approved by the Swedish National Board for Laboratory Animals (N138/14) and performed in accordance with the European Community Council Directive.

### Antigen recall experiments

After euthanizing the mice, draining lymph nodes were harvested, and draining lymph node cells were isolated by manual dissociation by passing the dissected lymph nodes through a 40-μm cell strainer. Cells were subsequently washed in PBS before being reconstituted in RPMI 1640 medium supplemented with 10% fetal bovine serum, 1% l-glutamine, 1% penicillin-streptomycin, 1% pyruvic acid (all Sigma-Aldrich), and 50 mM 2-mercapthoethanol (Gibco BRL). Cells were seeded in U-bottom plates at 0.2 × 10^6^ cells per well before adding an array of antigen beads at a concentration of 10 beads per cell. One well was kept without stimuli as a background control. A similar experiment was also made with soluble antigen, with stimulation of antigen (20 μg/ml). After a 72-hour incubation, the cells were moved to a V-bottom plate and restimulated with phorbol 12-myristate 13-acetate (50 ng/ml; Sigma-Aldrich) and ionomycin (1 μg/ml; Sigma-Aldrich) for 5 hours in the presence of brefeldin A (GolgiPlug) (1 μl/ml; BD Biosciences). Cells were subsequently stained with the following antibodies: CD3–fluorescein isothiocyanate, CD4 PE/Dazzle, Ki67-BV421 (all from BD Biosciences), and IFNγ-allophycocyanin (eBioscience). LIVE/DEAD Fixable Near-Infrared Dead Cell Stain (Invitrogen, L34976) was used to exclude dead cells. Intracellular/intranuclear staining was performed after permeabilization using a fixation/permeabilization kit (eBioscience). The results were collected using a Gallios flow cytometer (Beckman Coulter) and analyzed using Kaluza software (Beckman Coulter). Each biological replicate’s background response (NB) was subtracted from antigen responses before analysis and are presented as Δcounts.

### Statistical analysis

The suspension bead array MFI data were adjusted for each individual’s background responses (see “Suspension bead array” section for more details) and corrected for assay plate before statistical analysis. ORs were calculated using the Baptista-Pike method, with Fisher’s exact test for *P* values with Holm-Sidak correction for multiple comparisons. Crude ORs are reported. A threshold for positive response was created on the basis of the whole cohort’s response to the tested negative peptides (threshold at 99.9th percentile response, _Per33_MFI of 2484.85). Nonparametric statistical analyses were used except for the analysis of animal experiments. Wilcoxon signed-rank tests with Holm-Sidak correction for multiple comparisons were used for comparing the antibody blocking by analyzing the fold change compared to baseline of the blocked responses. For the comparison of FluoroSpot responses, two-tailed nonparametric Kruskal-Wallis test was used, with Dunn’s multiple comparisons test to correct for the multiple groups. Similarly, correlations were analyzed with a two-tailed nonparametric Spearman correlation with Holm-Sidak correction for multiple comparisons. Two- and one-way analyses of variance (ANOVAs) with Tukey’s multiple comparisons test were used for analysis of mouse antigen recall experiments. Calculations of threshold responses and sorting of data were performed in Excel (Microsoft Office, Microsoft), and statistical analyses were performed in Prism v.9 (GraphPad).

## Supplementary Materials

### This PDF file includes:

Figs. S1 to S13 Tables S1 to S4 Legend for data S1 References

## Notes

View/request a protocol for this paper from *Bio-protocol*.
