# Supplementary material for: Cross-reactive EBNA1 immunity targets alpha-crystallin B and is associated with multiple sclerosis
Source: Sci Adv. 2023 May 17;9(20):eadg3032. doi: 10.1126/sciadv.adg3032 (PMC10191428; doi:10.1126/sciadv.adg3032)
Supplement: Supplementary file 1 — Figs. S1 to S13 Tables S1 to S4 Legend for data S1 References [file sciadv.adg3032_sm.pdf]

Science Advances
AAAS

Supplementary Materials for

Cross-reactive EBNA1 immunity targets alpha-crystallin B and is associated with multiple sclerosis

Olivia G. Thomas *et al.*

Corresponding author: Olivia G. Thomas, olivia.thomas@ki.se

Sci. Adv. **9**, eadg3032 (2023)
DOI: 10.1126/sciadv.adg3032

The PDF file includes:

Figs. S1 to S13
Tables S1 to S4
Legend for data S1
References

Other Supplementary Material for this manuscript includes the following:

Data S1

Supplementary

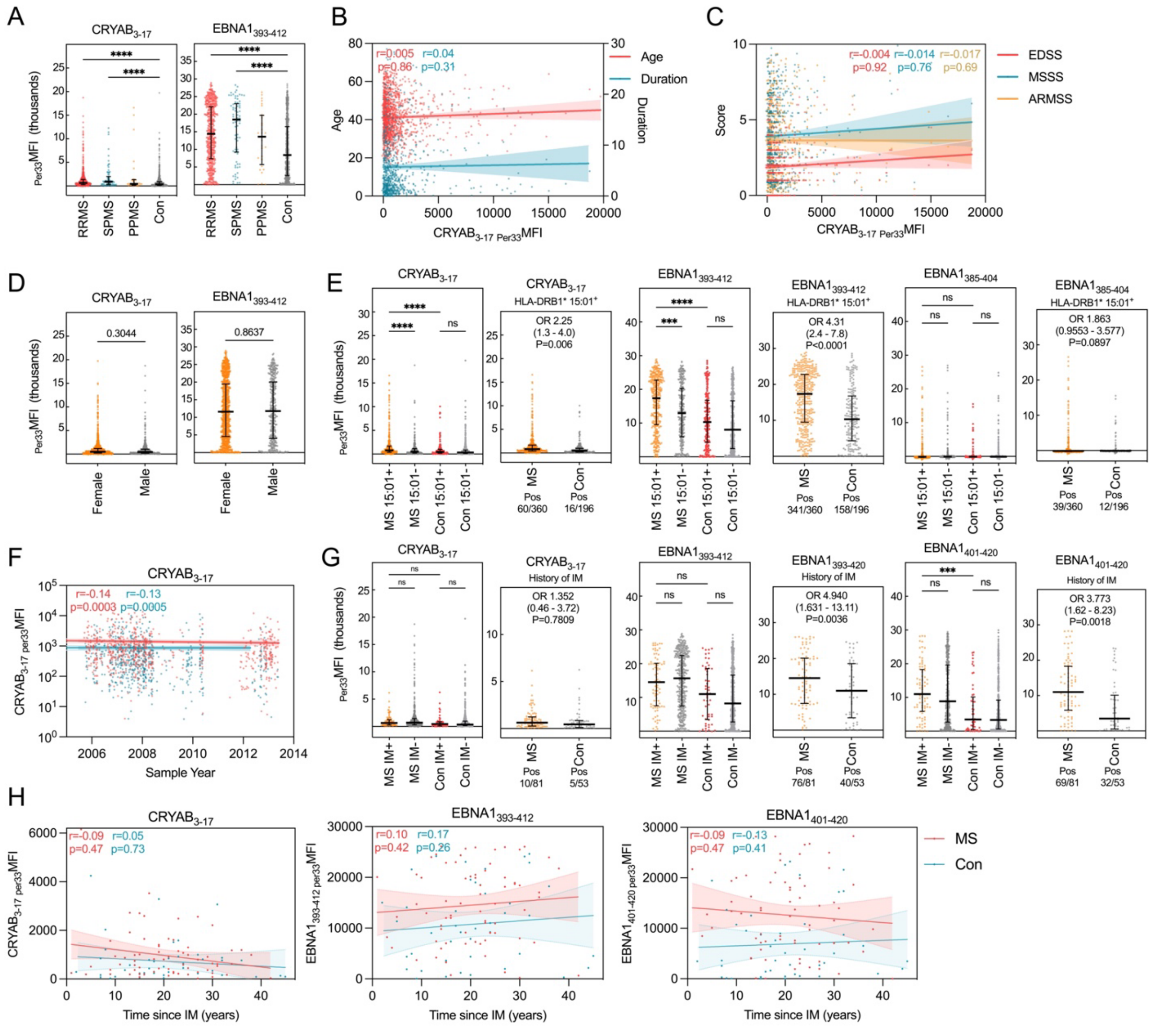

Supplementary Figure 1. CRYAB-reactivity in patient subgroups. A) Antibody reactivity (from Figure 1) stratified for disease status: relapsing-remitting MS (RRMS, n=590), secondary progressive MS (SPMS, n=75) and primary progressive MS (PPMS, including progressive-relapsing MS, n=28). **B**) Correlation between CRYAB reactivity and age (red, plotted on left Y-axis) and disease duration (blue, plotted on right y-axis). **C**) Correlation between CRYAB reactivity and EDSS (red), MSSS (blue) and ARMSS (orange) (plotted on left Y-axis). **D**) Anti-CRYAB and anti-EBNA1 antibodies in females and males. **E**) Leftmost panel: CRYAB$_{3-17}$ antibody reactivity (from Figure 1) in all donors stratified for HLA-DRB1*15:01 status (MS HLA-DRB1*15:01+ n=360 (orange), MS HLA-DRB1*15:01- n=289 (gray), Con HLA-DRB1*15:01+ n=196 (red), Con HLA-DRB1*15:01- n=479 (gray)). Center left panel: CRYAB$_{3-17}$ antibody reactivity in HLA-DRB1*15:01+ donors only (MS 60/360 CRYAB$_{3-17}$+ (orange), Con 16/196 CRYAB$_{3-17}$+ (gray)). Center right panel: EBNA1$_{393-412}$ antibody

reactivity stratified for HLA-DRB1*15:01 status (n values as in leftmost panel). Rightmost panel: $EBNA1_{393-412}$ antibody reactivity in HLA-DRB1*15:01+ donors only (MS 341/360 $EBNA1_{393-412}$+ (orange), Con 158/196 $EBNA1_{393-412}$+ (gray)). **F**) Correlation between $CRYAB_{3-17}$ $_{per33}$MFI (y-axis) and sample year (x-axis). **G**) Antibody reactivity stratified for history of IM (MS IM+ n=81 (red), MS IM- n=407 (gray), Con IM+ n=53 (orange), Con IM- n=518 (gray)). Leftmost panel: $CRYAB_{3-17}$. Middle left panel: $CRYAB_{3-17}$ antibody reactivity in IM+ individuals only. Middle panel: $EBNA1_{393-412}$ antibody responses stratified for history of IM. Middle right panel: $EBNA1_{401-420}$ stratified for history of IM. Rightmost panel: $EBNA1_{401-420}$ antibody responses in IM+ individuals only. **H**) Correlation of antibody responses ($_{per33}$MFI, y-axis) in MS (red, n=70) and Con (blue, n=43) with time since self-reported IM (years, x-axis) for $CRYAB_{3-17}$ (left panel), $EBNA1_{393-412}$ (middle panel) and $EBNA1_{401-420}$ (right panel). For comparison of antibody titres between two groups: P-values were calculated using a Mann-Whitney U-test. For comparison of antibody titres between multiple groups: P-values were calculated using a Kruskal-Wallis test with Dunn's correction for multiple comparisons and indicated where significant. Each dot represents one individual, staples denote median and IQR. For correlations: r- and P-values were calculated using Spearman correlation. Lines and shaded areas denote linear regression slopes and the 95%CI of slopes. ORs of MS vs. Con positive responses defined as >99.9^{th} percentile of negative control peptide responses. ORs were calculated using the Baptista-Pike method with Fisher's exact test for P-values and Holm-Sidak correction for multiple comparisons. *p<0.05. **p<0.01. ***p<0.001. ****p<0.0001.

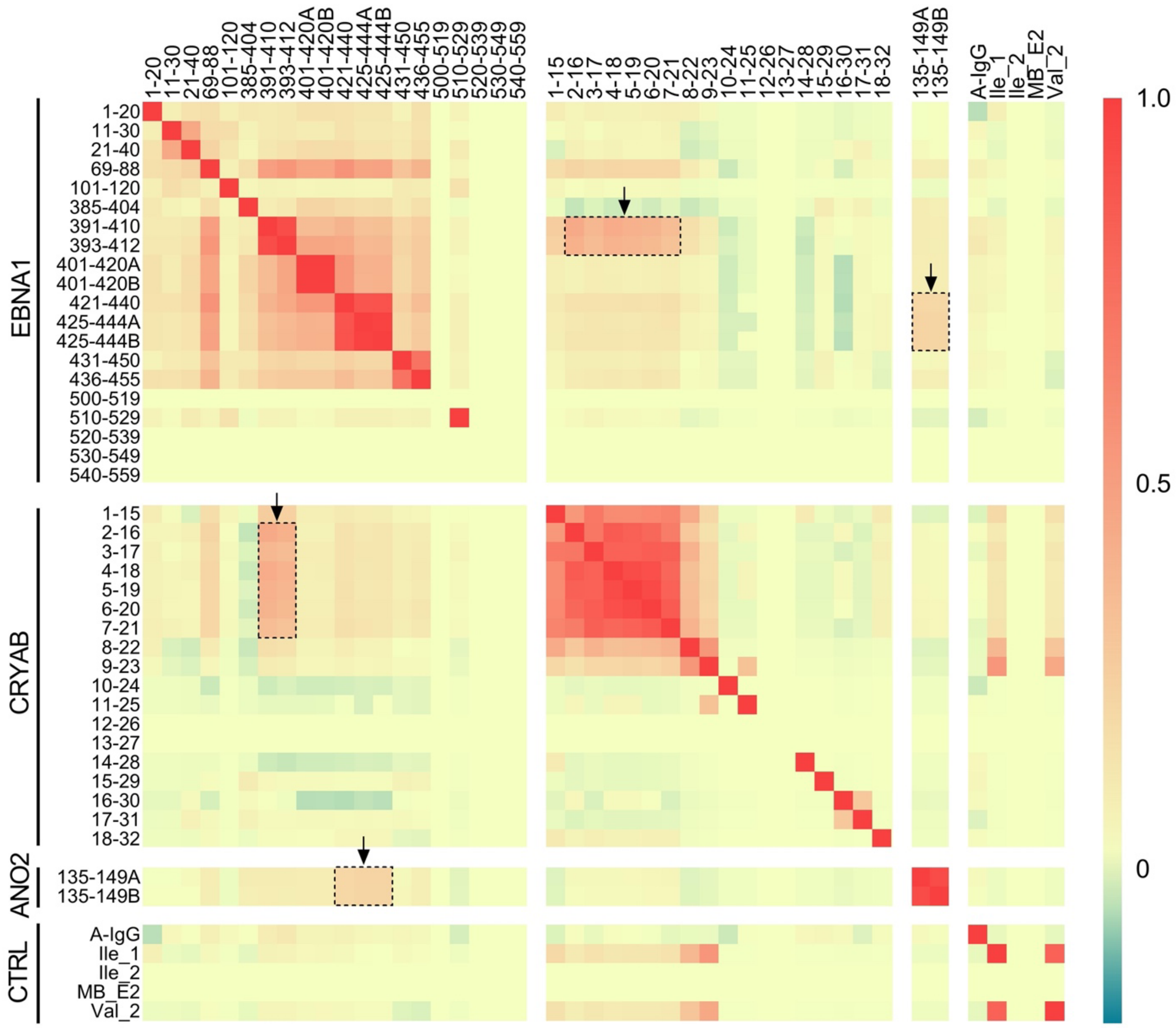

Supplementary Figure 2. Correlation matrix of all tested peptides. Correlation matrix based on positive responses, excluding assay background below the 2484.85 per33MFI threshold. The scale denotes non-parametric Spearman r-value. Unobtained r-values (no available comparisons) are plotted as r=0. Ctrl indicates assay controls.

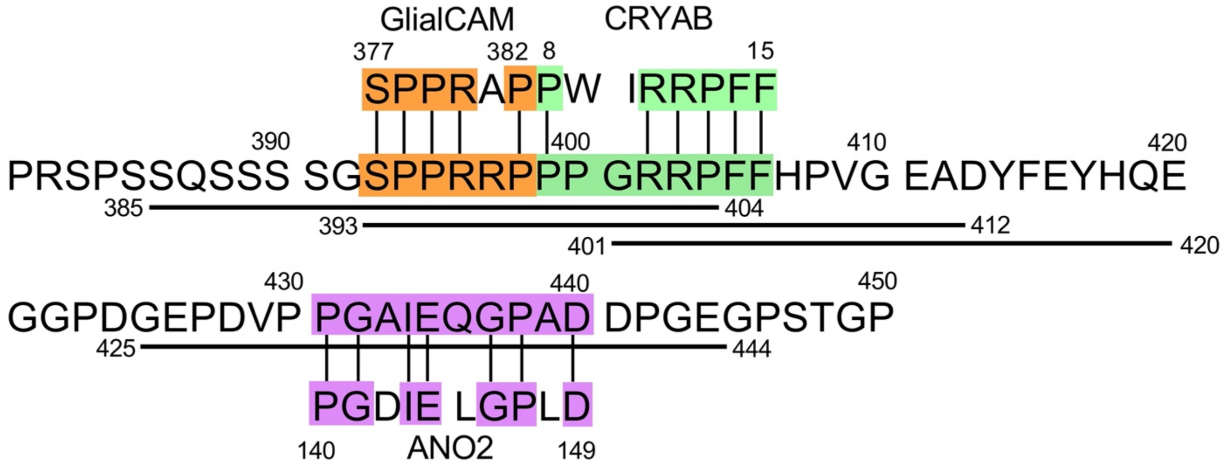

Supplementary Figure 3. EBNA1 homologies to reported autoantigens. Amino acid sequence of $EBNA1_{381-450}$ with homologies to GlialCAM (orange), CRYAB (green) and ANO2 (magenta). Black lines under the amino acid sequence denote the tested EBNA1 peptides with odds ratios reported in Figure 1.

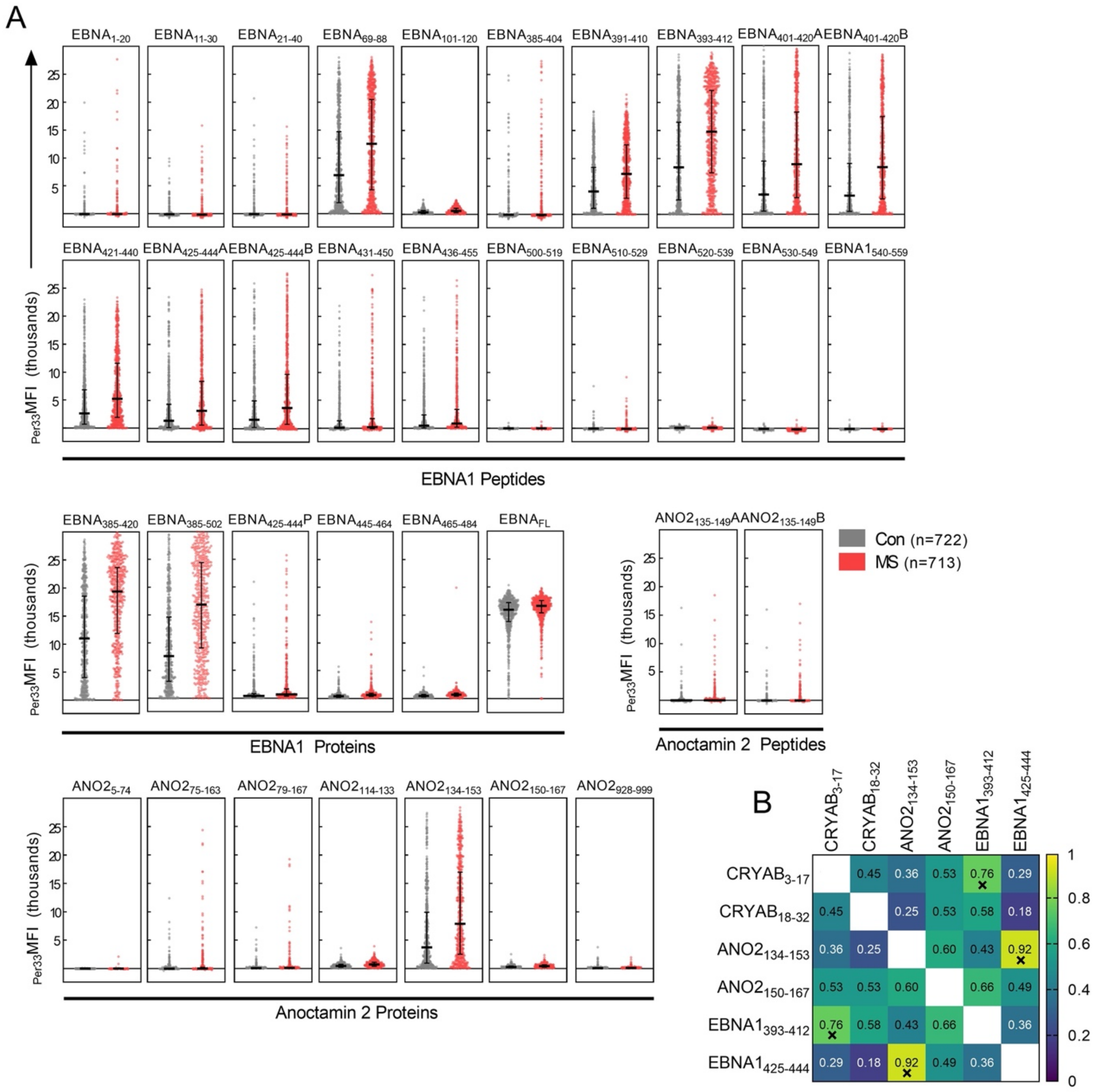

Supplementary Figure 4. IgG against EBNA1 and ANO2. Suspension bead array of IgG reactivity, all tested EBNA1 and ANO2 variants related to Figure 1. **A**) Background-adjusted mean fluorescent intensity ($_{Per33}$MFI) values of all tested individuals against EBNA1 peptides and proteins plus ANO2 peptides and proteins. Each dot represents one individual and staples represent the median and IQR. **B**) Correlation matrix of CRYAB and ANO2 peptides both with and without sequence homology to EBNA1. Two corresponding EBNA1 peptides containing homology to autoantigens marked with an X ($CRYAB_{3\text{-}17}$ and $EBNA_{393\text{-}412}$; $ANO2_{134\text{-}153}$ and $EBNA1_{425\text{-}444}$). Numbers and scale denote Spearman r-value. Full correlation matrix is available in Supplementary Figure 2.

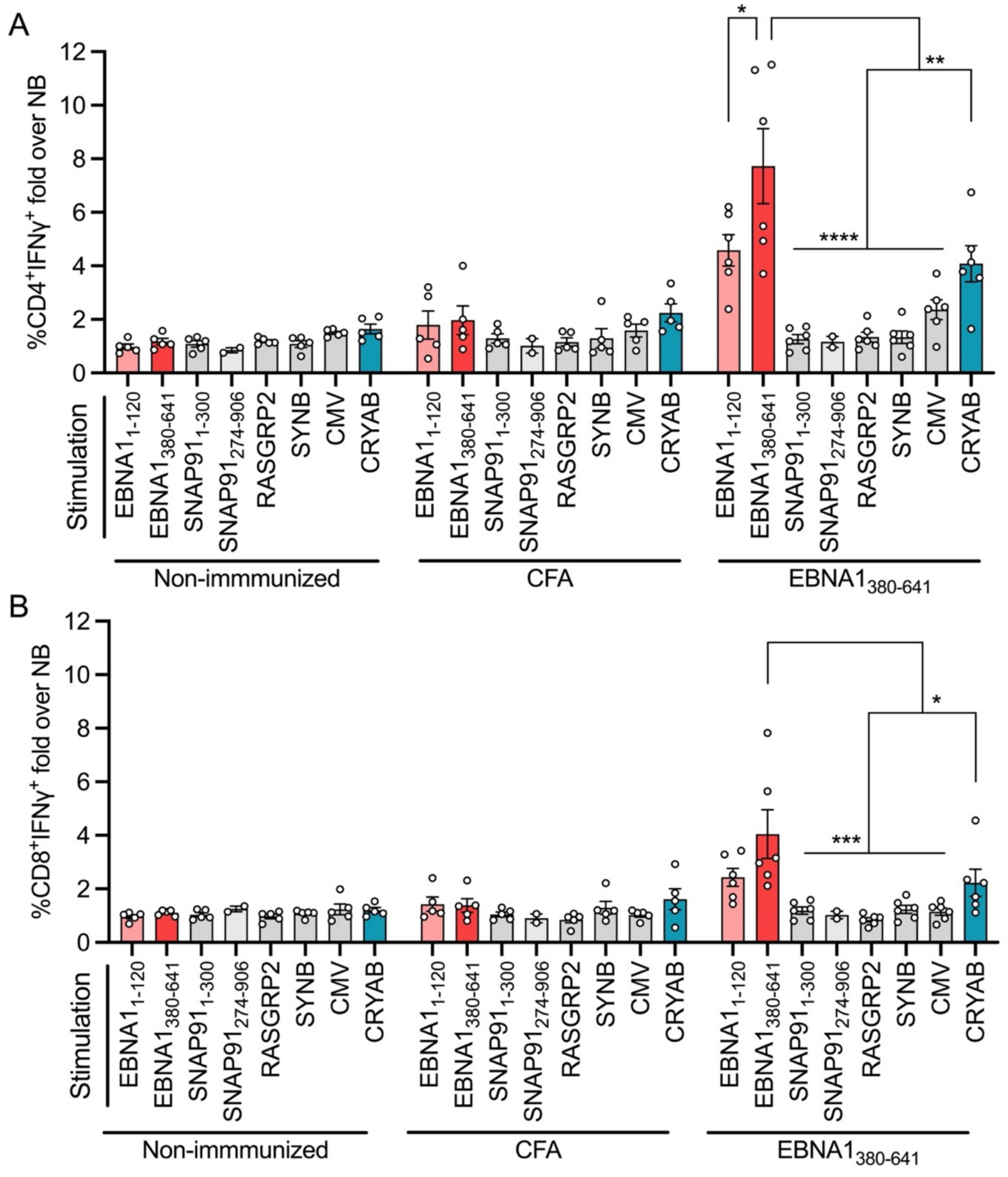

Supplementary Figure 5. Recall stimulations of draining lymph node cells from control- or EBNA1-immunized mice. Related to Figure 3. The data presented is compiled from two separate experiments where draining lymph node cells from non-immunized mice (n=5) or mice immunized with CFA (n=5) or EBNA1$_{380-641}$ (n=6) were restimulated with bead-bound antigen *in vitro* on day 10 and analyzed by intracellular cytokine staining and flow cytometry. **A)** $CD4^+$ T cells responding to bead-bound antigens. Data is presented as the fold change of $\%CD3^+CD4^+IFN\gamma^+$ over the naked bead (NB) negative stimulation control. **B)** Responding $CD8^+$ T cells after stimulation with bead-bound antigens. Data is presented as the fold change of $\%CD3^+CD8^+IFN\gamma^+$ over NB negative control. Bars and staples denote mean ± SEM. Each dot represents a biological replicate. P-values were calculated using a two-way ANOVA with Tukey's multiple comparisons test, comparing each different re-stimulation within the immunization group and indicated where significant. P-

values were calculated using a one-way ANOVA with Tukey's multiple comparisons test. *$p<0.05$. **$p<0.01$. ***$p<0.001$. ****$p<0.0001$. SNAP91: synaptosomal-associated protein 91. RASGRP2: RAS-guanyl releasing protein 2. SYNB: Synuclein beta.

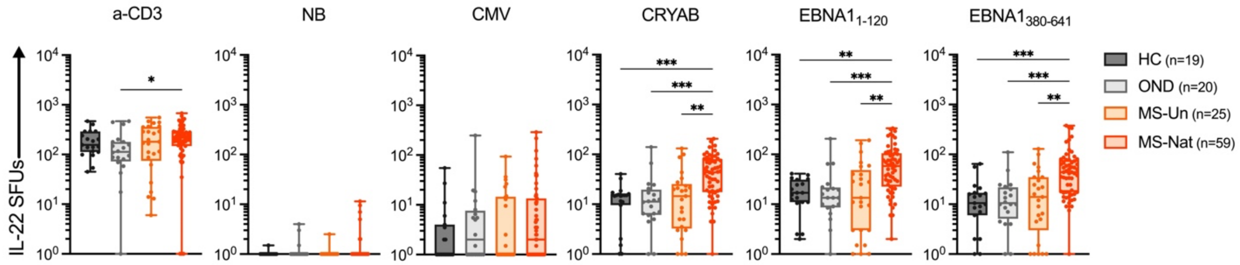

Supplementary Figure 6. IL-22 FluoroSpot. Related to Figure 4. Number of IL-22 spot forming units (SFUs) in a FluoroSpot assay after control, CRYAB and EBNA1 stimulations. SFUs <1 are plotted as 1. Boxes represent median ± IQR. P-values were calculated with a non-parametric two-tailed Kruskal-Wallis test with Dunn's multiple comparison test. Each group was compared with every other and p-values are indicated where significant. *p<0.05. **p<0.01. ***p<0.001. ****p<0.0001. CMV: Cytomegalovirus. NB: naked beads. MS-Un: untreated persons with MS. MS-Nat: Natalizumab-treated persons with MS. HC: healthy controls. OND: individuals with other neurological disease.

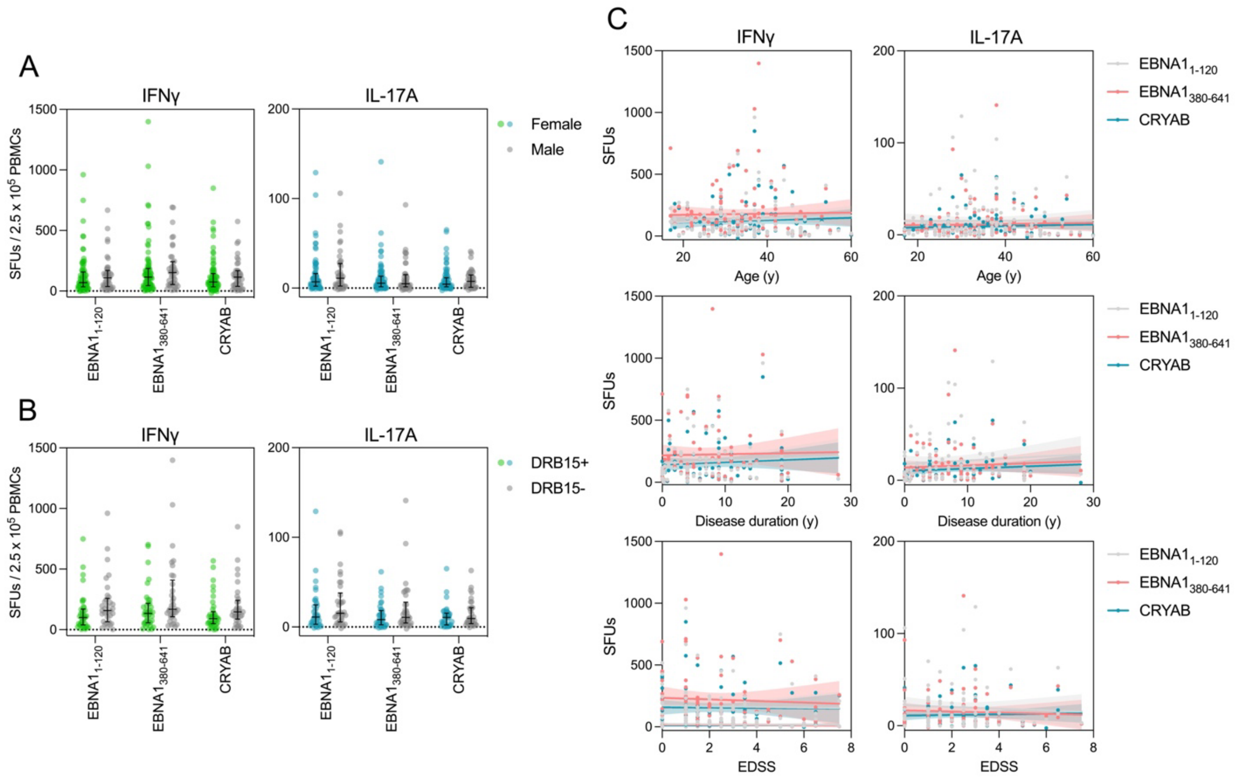

Supplementary Figure 7. Anti-CRYAB and EBNA1 responses relation to clinical characteristics. A, B) CRYAB and EBNA1 T cell responses (from Figure 4) stratified based on sex (**A**) and HLA-DRB*15:01 status (**B**). Statistical differences were calculated using the Mann-Whitney U test, no significances were found. **C**) Correlations between CRYAB and EBNA1 IFNγ responses (left-hand panels) and IL-17A responses (right-hand panels). Each dot represents one individual. The lines and shaded areas represent linear regression slopes with 95% confidence interval respectively. Correlations were calculated using non-parametric Spearman r and no significant correlations were found.

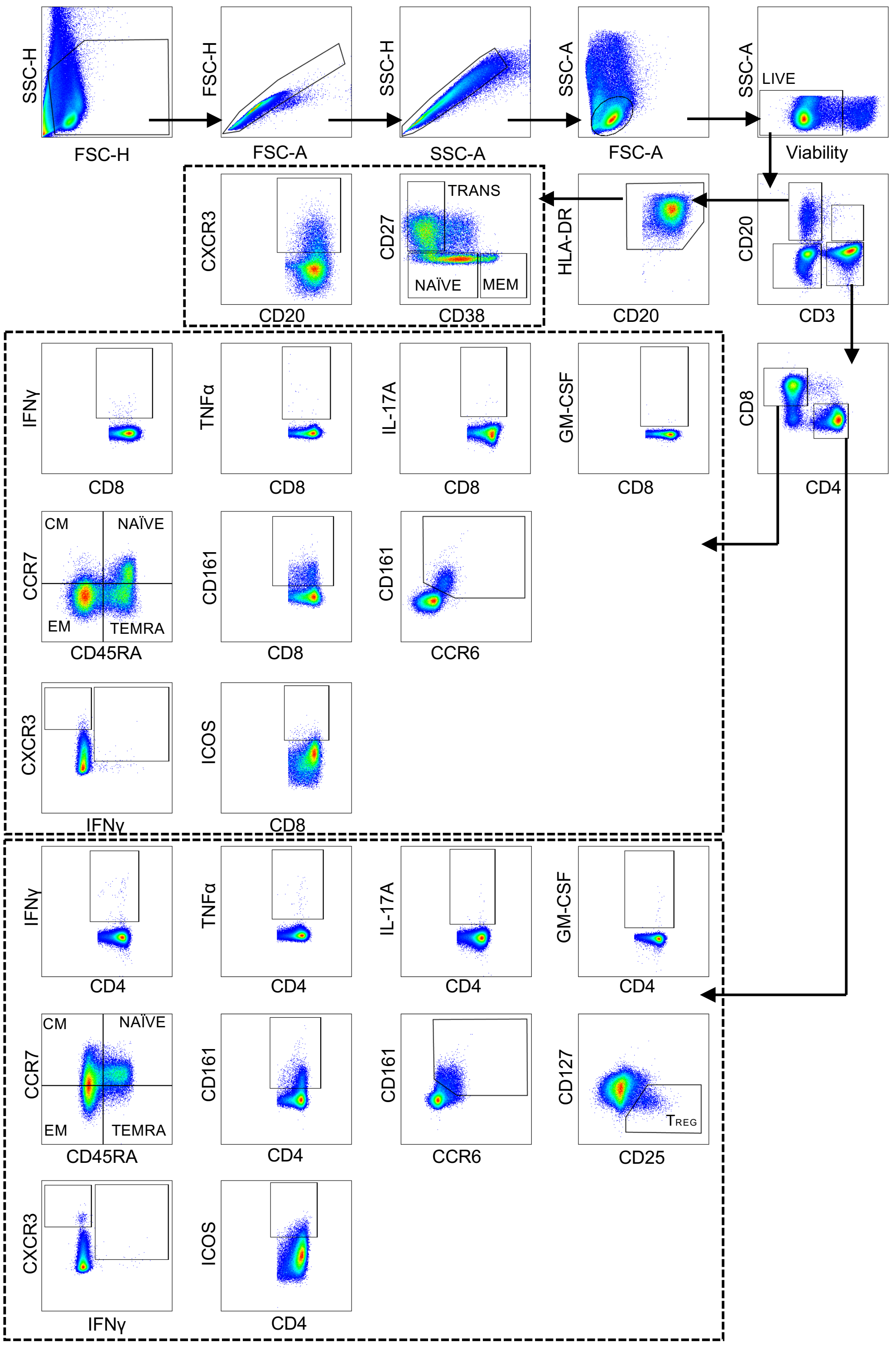

Supplementary Figure 8. Spectral flow cytometry gating strategy. Flow cytometry analysis of PBMC using intracellular cytokine staining after antigen bead stimulation. Representative plots demonstrating gating strategy for populations to which data is shown. SSC, side scatter; FSC, forward scatter.

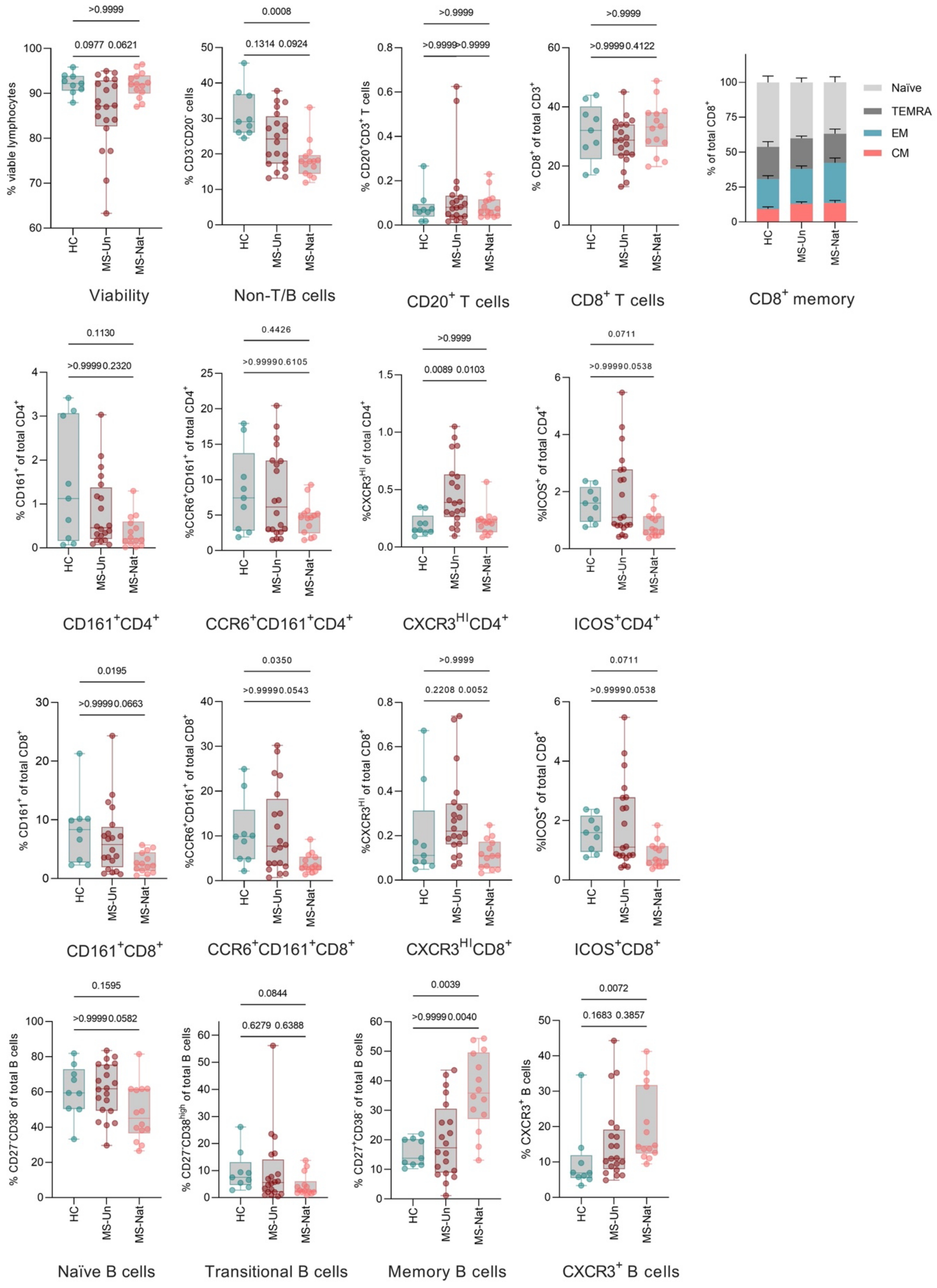

Supplementary Figure 9. Lymphocyte subsets in bulk PBMCs. Overall distribution of

different lymphocyte subsets. Related to Figure 5. P-values were calculated using the Kruskal-Wallis test with Dunn's multiple comparisons test.

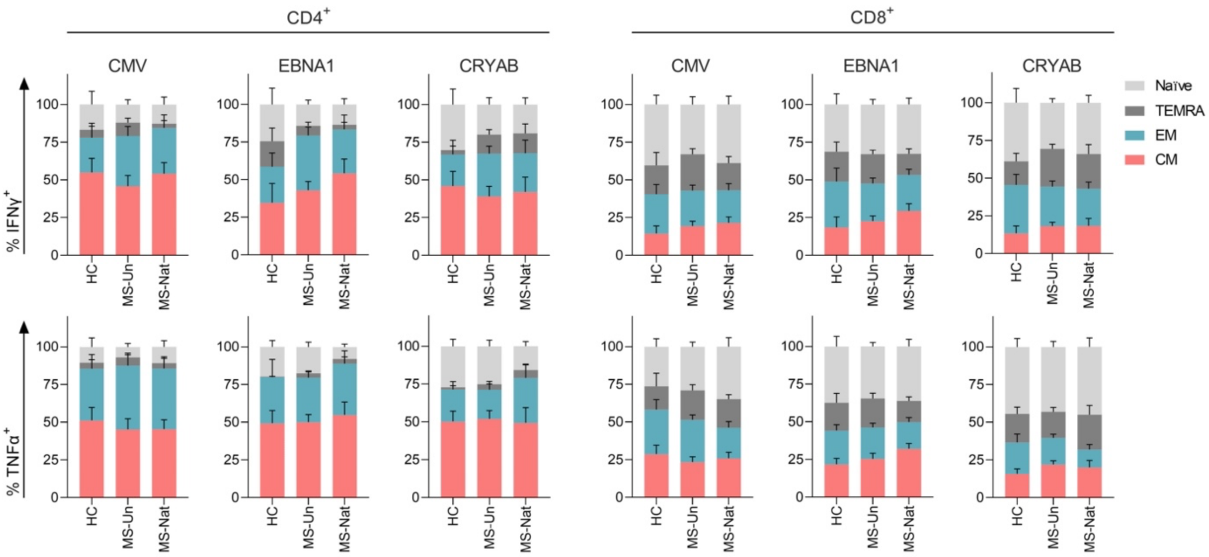

Supplementary Figure 10. Phenotype of antigen-responding T cells. Full dataset related to Figure 5D. Defined as: central memory (CM, $CCR7^{+}CD45RA^{-}$), effector memory (EM, $CCR7^{-}CD45RA^{-}$), terminally-differentiated EM ($CCR7^{-}CD45RA^{+}$) and naïve ($CCR7^{+}CD45RA^{+}$). Bars and staples denote the mean and SEM respectively.

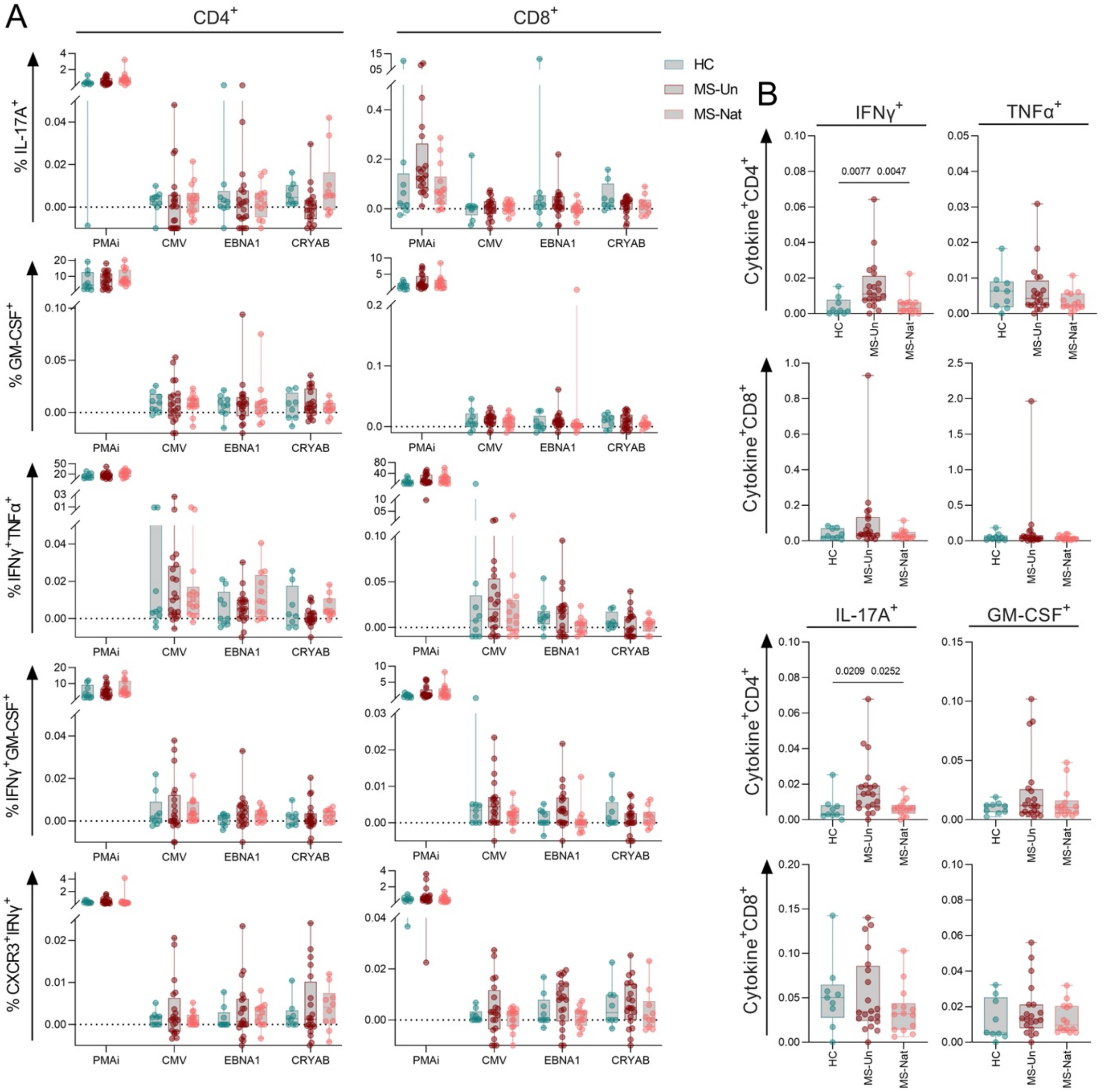

Supplementary Figure 11. Intracellular cytokine staining of antigen-stimulated T cells. A) Cytokine responses in CD4+ and CD8+ T cells after stimulation with antigen beads. **B**) Background cytokine responses (naked bead stimulation) plotted as raw values. NB. All other intracellular cytokine staining graphs (Figure 5 and Supplementary Figure 10A) use data which has been adjusted by subtracting this background response from targets (% cytokine positive cells after antigen stimulation minus % cytokine positive cells after naked bead stimulation). P-values were calculated using a Kruskal-Wallis test with Dunn's multiple comparisons test.

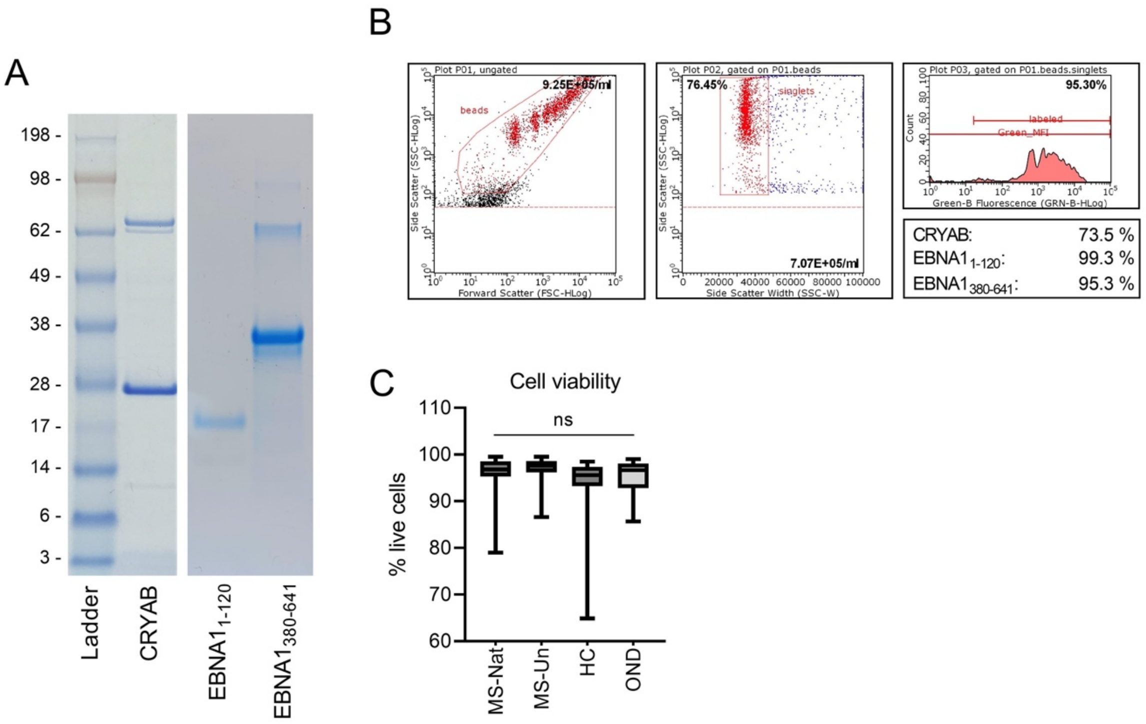

Supplementary Figure 12. FluoroSpot QC. A) SDS-page gel of purified CRYAB, EBNA1$_{1-120}$ and EBNA1$_{380-640}$. Image spliced from two different gels run with the same reference ladder, placing of righthand gel in reference the ladder is kept consistent. Numbers indicate ladder step size (kDa). Linear color correction and brightness adjustment have been made to original image, irrelevant lanes have been cropped out. **B**) Antigen bead coupling quality control using flow cytometry. Representative plots show gating strategy. **C**) Viability after thawing of cryopreserved PBMCs. Statistical differences were calculated using a one-way ANOVA.

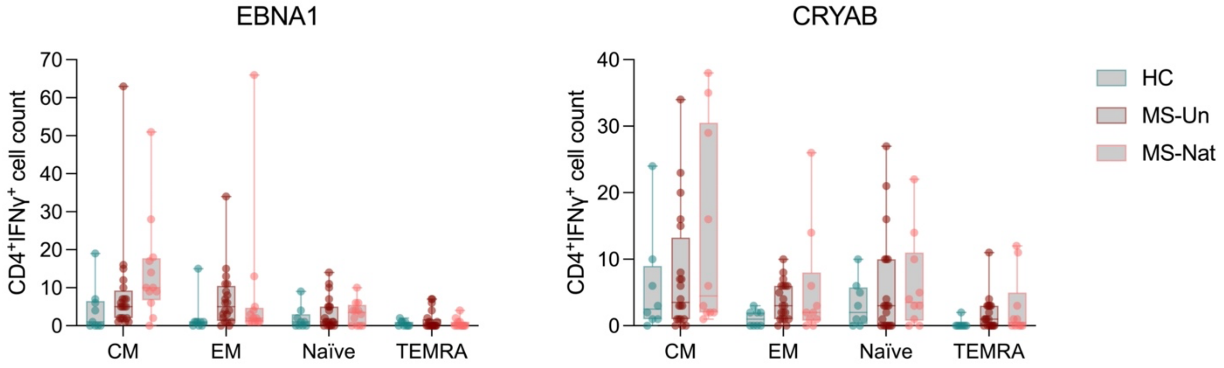

Supplementary Figure 13. Cell counts used to evaluate memory phenotype of responding CD4$^+$IFNγ$^+$ T cells by flow cytometry and intracellular cytokine staining. Cell counts related to Figure 5D.

Supplementary Table 1. CRYAB-fragments

Name	Source	Amino acid sequence*
CRYAB$_{1-15}$		MDIAIHH**PWIRRPFF**
CRYAB$_{2-16}$		DIAIHH**PWIRRPFF**P
CRYAB$_{3-17}$		IAIHH**PWIRRPFF**PF
CRYAB$_{4-18}$		AIHH**PWIRRPFF**PFH
CRYAB$_{5-19}$		IHH**PWIRRPFF**PFHS
CRYAB$_{6-20}$		HH**PWIRRPFF**PFHSP
CRYAB$_{7-21}$		H**PWIRRPFF**PFHSPS
CRYAB$_{8-22}$		**PWIRRPFF**PFHSPSR
CRYAB$_{9-23}$	Synthesized – PEPscreen, Sigma-Aldrich	**WIRRPFF**PFHSPSRL
CRYAB$_{10-24}$		**IRRPFF**PFHSPSRLF
CRYAB$_{11-25}$		**RRPFF**PFHSPSRLFD
CRYAB$_{12-26}$		**RPFF**PFHSPSRLFDQ
CRYAB$_{13-27}$		**PFF**PFHSPSRLFDQF
CRYAB$_{14-28}$		**FF**PFHSPSRLFDQFF
CRYAB$_{15-29}$		**F**PFHSPSRLFDQFFG
CRYAB$_{16-30}$		PFHSPSRLFDQFFGE
CRYAB$_{17-31}$		FHSPSRLFDQFFGEH
CRYAB$_{18-32}$		HSPSRLFDQFFGEHL
CRYAB$_{12-99}$	Recombinant PrEST - HPRR2700063, Human Protein Atlas	**RPFF**PFHSPSRLFDQFFGEHLLESDLFPTSTSLSPFYLRPPSFLRAPSWFDTGLSEMRLEKDRFSVNLDVKHFSPEELKVKVLGDVIE
CRYAB$_{103-172}$	Recombinant PrEST - HPRR2700064, Human Protein Atlas	KHEERQDEHGFISREFHRKYRIPADVDPLTITSSLSSDGVLTVNGPRKQVSGPERTIPITREEKPAVTAA
CRYAB$_{121-172}$	Recombinant PrEST - HPRR3880249, Human Protein Atlas	KYRIPADVDPLTITSSLSSDGVLTVNGPRKQVSGPERTIPITREEKPAVTAA
CRYAB$_{1-175}$	Recombinant full-length protein – NBC1-18352, Novus Biologicals	MDIAIHH**PWIRRPFF**PFHSPSRLFDQFFGEHLLESDLFPTSTSLSPFYLRPPSFLRAPSWFDTGLSEMRLEKDRFSVNLDVKHFSPEELKVKVLGDVIEVHGKHEERQDEHGFISREFHRKYRIPADVDPLTITSSLSSDGVLTVNGPRKQVSGPERTIPITREEKPAVTAAPKK

PrEST: Protein epitope signature tag, Human Protein Atlas(*62*). *Amino acid sequences in **bold** denote EBNA1 sequence homology with core sequence underlined.

Supplementary Table 2. Odds ratios for multiple sclerosis with different CRYAB and EBNA1-reactivities.

Specificity	Positive patients (n/713, %)	Positive controls (n/722, %)	Odds Ratio	CI 95% Low bound	CI 95% High bound	P-value (corrected)
$CRYAB_{1-15}$	52 (7.3)	33 (4.6)	1.64	1.04	2.57	0.50894
**$CRYAB_{2-16}$**	**197 (27.6)**	**122 (16.9)**	**1.88**	**1.45**	**2.43**	**$3.58E^{-05}$**
**$CRYAB_{3-17}$**	**95 (13.2)**	**52 (7.2)**	**1.98**	**1.40**	**2.82**	**0.00413**
**$CRYAB_{4-18}$**	**147 (20.6)**	**89 (12.3)**	**1.85**	**1.39**	**2.46**	**0.00067**
**$CRYAB_{5-19}$**	**132 (18.5)**	**83 (11.5)**	**1.75**	**1.31**	**2.35**	**0.00501**
**$CRYAB_{6-20}$**	**124 (17.4)**	**72 (10.0)**	**1.90**	**1.40**	**2.60**	**0.00113**
**$CRYAB_{7-21}$**	**100 (14.9)**	**58 (8.0)**	**1.87**	**1.33**	**2.61**	**0.00814**
$CRYAB_{8-22}$	14 (2.0)	8 (1.1)	1.79	0.76	4.06	0.97997
$CRYAB_{9-23}$	6 (0.8)	4 (0.6)	1.52	0.41	4.80	0.99997
$CRYAB_{10-24}$	1 (0.1)	0	Infinity	0.11	Infinity	0.99997
$CRYAB_{11-25}$	1 (0.1)	0	Infinity	0.11	Infinity	0.99997
$CRYAB_{12-26}$	0	0	-	-	-	1
$CRYAB_{13-27}$	0	0	-	-	-	1
$CRYAB_{14-28}$	1 (0.1)	0	Infinity	0.11	Infinity	0.99997
$CRYAB_{15-29}$	1 (0.1)	0	Infinity	0.11	Infinity	0.99997
$CRYAB_{16-30}$	2 (0.3)	4 (0.6)	0.50	0.10	2.17	0.99997
$CRYAB_{17-31}$	1 (0.1)	1 (0.1)	1.0	0.05	19.26	1
$CRYAB_{18-32}$	1 (0.1)	0	Infinity	0.11	Infinity	0.99997
$CRYAB_{12-99}$	0	0	-	-	-	1
$CRYAB_{103-172}$	2 (0.3)	0	Infinity	0.11	Infinity	0.98573
$CRYAB_{121-172}$	3 (0.4)	0	Infinity	0.11	Infinity	0.90806
$CRYAB_{1-175}$	3 (0.4)	0	Infinity	0.11	Infinity	0.90806
$EBNA1_{385-404}$	61 (8.6)	48 (6.6)	1.31	0.88	1.95	0.97994
**$EBNA1_{393-412}$**	**642 (90.0)**	**545 (75.5)**	**2.92**	**2.17**	**3.94**	**$<1E^{-14}$**
**$EBNA1_{401-420}$**	**544 (76.3)**	**398 (55.1)**	**2.62**	**2.10**	**3.28**	**$<1E^{-14}$**
**$EBNA1_{425-444}$**	**425 (59.6)**	**301 (41.7)**	**2.06**	**1.67**	**2.54**	**$3.72E^{-10}$**
**$EBNA1_{high}$**	**94 (13.2)**	**31 (4.3)**	**3.39**	**2.22**	**5.10**	**$5.30E^{-08}$**
**$CRYAB_{neg}$+$EBNA1_{high}$**	**60 (8.4)**	**27 (3.7)**	**2.37**	**1.51**	**3.78**	**0.00524**
**$CRYAB_{pos}$+$EBNA1_{high}$**	**34 (4.8)**	**4 (0.6)**	**8.99**	**3.39**	**23.76**	**$6.09E^{-06}$**
Ile_1	2 (0.3)	1 (0.1)	2.028	0.2352	29.43	0.99996
Ile_2	0	0	-	-	-	1
Val_2	1 (0.1)	1 (0.1)	1.0	0.05	19.26	1
MB_E2	0	0	-	-	-	1

Supplementary Table 3. Cohort characteristics

	Antibody Cohort		T cell Cohort			
	**MS**	**HC**	**MS-Nat**	**MS-Un**	**HC**	**OND**
Size (n)	713	722	59	25	19	20
Age (years)	39.9 ± 10.2 (17-67)	42.6 ± 10.4 (17-68)	36.2 ± 10.0 (17-61)	37.1 ± 11 (17-60)	33.6 ± 6.7 (24-48)	28.4 ± 9.7 (18-57)
Sex (female)	70.3 %	74.9 %	71.2 %	76.9 %	63.2 %	45.0 %
HLA-DRB1*15:01 (n)	360/649 (55.5%)	196/673(29.1 %)	27 / 54 (50 %)	-	2 / 8 (25 %)	9 / 11 (81.8 %)
EDSS (score)	1.75±1.5	-	2.3 ± 1.7 (0.0–7.5)	2.1 ± 1.6 (0.0–6)	-	-
Disease Duration (years)	5.7 ± 6.1 (0-33)	-	8.5 ± 5.4 (1-20)	5.2 ± 7.8 (0-28)	-	-
History of IM (n, +/-/NA)	81/407/225	53/518/151	-	-	-	-

± denotes SD. Range or percentage of whole in brackets, apart from EDSS where this is presented as median ± IQR as this data is not normally distributed. n / N denotes the number of individuals for which data was available if it was not the whole cohort. HC: Healthy Controls. MS-Nat: Natalizumab-treated persons with MS. MS-Un: Untreated persons with MS. OND: Persons with other neurological disease. EDSS: Expanded Disability Status Scale. History of IM (self-reported), NA (number of donors for which information was not available).

Supplementary Table 4. Spectral flow cytometry reagents.

Cocktail	Marker	Fluorophore	Clone	Catalogue no.	Vendor
Intracellular	IL-17A	BV421	BL168	512322	BioLegend
Surface	CD20	cFluor V450	2H7	SKU R7-20016	Cytek
Surface	CD69	BV480	FN50	747519	BD
Surface	CD8	BV510	SK1	563919	BD
Surface	CD45RA	BV570	HI300	304131	BioLegend
Surface	CCR6	BV605	11A9	562724	BD
Surface	CD27	BV650	M-T271	564894	BD
Surface	Ki67	BV711	B56	563755	BD
Surface	CD161	BV750	DX12	746921	BD
Surface	CD40L	BV785	24-31	310842	BioLegend
Intracellular	TNFα	Alexa Fluor 488	MAb11	502915	BioLegend
Surface	CD3	Spark Blue 550	SK7	344851	BioLegend
Intracellular	IFNγ	BB700	B27	566394	BD
Surface	CCR7	PerCP-eFluor 710	3D12	46-1979-42	eBioscience
Surface	CD25	PE	BC96	302606	BioLegend
Surface	PD-1	PE/Dazzle 594	EH12.2H7	329940	BioLegend
Surface	ICOS	PE-Cy5	C398.4A	313560	BioLegend
Surface	CD127	PE-Cy5.5	eBioRDR5	35-1278-42	ThermoFisher Scientific
Surface	CXCR3	PE-Cy7	G025H7	353720	BioLegend
Intracellular	GM-CSF	APC	BVD2-21C11	502310	BioLegend
Surface	CD4	cFluor R685	SK3	SKU R7-20052	Cytek
Surface	CD38	cFluor R720	HIT2	SKU R7-20062	Cytek
Viability	Viability	ViaDye Red	-	SKU R7-60008	Cytek
Surface	HLA-DR	APC-Fire 750	L243	307658	BioLegend
Intracellular	IL-17A	BV421	BL168	512322	BioLegend

Other Supplementary Materials

adg3032_Data_S1.xlsx

Source data for Fig. 1-5 and Fig. S1, S2, S4, S5, S6, S7, S9, S10, S11, S12, S13

REFERENCES AND NOTES

1. C. A. Dendrou, L. Fugger, M. A. Friese, Immunopathology of multiple sclerosis. *Nat. Rev. Immunol.* **15**, 545–558 (2015).

2. International Multiple Sclerosis Genetics Consortium; Wellcome Trust Case Control Consortium 2, S. Sawcer, G. Hellenthal, M. Pirinen, C. C. A. Spencer, N. A. Patsopoulos, L. Moutsianas, A. Dilthey, Z. Su, C. Freeman, S. E. Hunt, S. Edkins, E. Gray, D. R. Booth, S. C. Potter, A. Goris, G. Band, A. B. Oturai, A. Strange, J. Saarela, C. Bellenguez, B. Fontaine, M. Gillman, B. Hemmer, R. Gwilliam, F. Zipp, A. Jayakumar, R. Martin, S. Leslie, S. Hawkins, E. Giannoulatou, S. D'alfonso, H. Blackburn, F. M. Boneschi, J. Liddle, H. F. Harbo, M. L. Perez, A. Spurkland, M. J. Waller, M. P. Mycko, M. Ricketts, M. Comabella, N. Hammond, I. Kockum, O. T. Mc Cann, M. Ban, P. Whittaker, A. Kemppinen, P. Weston, C. Hawkins, S. Widaa, J. Zajicek, S. Dronov, N. Robertson, S. J. Bumpstead, L. F. Barcellos, R. Ravindrarajah, R. Abraham, L. Alfredsson, K. Ardlie, C. Aubin, A. Baker, K. Baker, S. E. Baranzini, L. Bergamaschi, R. Bergamaschi, A. Bernstein, A. Berthele, M. Boggild, J. P. Bradfield, D. Brassat, S. A. Broadley, D. Buck, H. Butzkueven, R. Capra, W. M. Carroll, P. Cavalla, E. G. Celius, S. Cepok, R. Chiavacci, F. Clerget-Darpoux, K. Clysters, G. Comi, M. Cossburn, I. Cournu-Rebeix, M. B. Cox, W. Cozen, B. A. C. Cree, A. H. Cross, D. Cusi, M. J. Daly, E. Davis, P. I. W. de Bakker, M. Debouverie, M. B. D'hooghe, K. Dixon, R. Dobosi, B. Dubois, D. Ellinghaus, I. Elovaara, F. Esposito, C. Fontenille, S. Foote, A. Franke, D. Galimberti, A. Ghezzi, J. Glessner, R. Gomez, O. Gout, C. Graham, S. F. A. Grant, F. R. Guerini, H. Hakonarson, P. Hall, A. Hamsten, H.-P. Hartung, R. N. Heard, S. Heath, J. Hobart, M. Hoshi, C. Infante-Duarte, G. Ingram, W. Ingram, T. Islam, M. Jagodic, M. Kabesch, A. G. Kermode, T. J. Kilpatrick, C. Kim, N. Klopp, K. Koivisto, M. Larsson, M. Lathrop, J. S. Lechner-Scott, M. A. Leone, V. Leppä, U. Liljedahl, I. L. Bomfim, R. R. Lincoln, J. Link, J. Liu, A. R. Lorentzen, S. Lupoli, F. Macciardi, T. Mack, M. Marriott, V. Martinelli, D. Mason, J. L. McCauley, F. Mentch, I.-L. Mero, T. Mihalova, X. Montalban, J. Mottershead, K.-M. Myhr, P. Naldi, W. Ollier, A. Page, A. Palotie, J. Pelletier, L. Piccio, T. Pickersgill, F. Piehl, S. Pobywajlo, H. L. Quach, P. P. Ramsay, M. Reunanen, R. Reynolds, J. D. Rioux, M. Rodegher, S. Roesner, J. P. Rubio, I.-M. Rückert, M. Salvetti, E. Salvi, A. Santaniello, C. A. Schaefer, S. Schreiber, C. Schulze, R. J. Scott, F. Sellebjerg, K. W. Selmaj, D. Sexton, L. Shen, B. Simms-Acuna, S. Skidmore, P. M. A. Sleiman, C. Smestad, P. S. Sørensen, H. B. Søndergaard, J. Stankovich, R. C. Strange, A.-M. Sulonen, E. Sundqvist, A.-C. Syvänen, F. Taddeo, B. Taylor, J. M. Blackwell, P. Tienari, E. Bramon, A. Tourbah, M. A. Brown, E.

Tronczynska, J. P. Casas, N. Tubridy, A. Corvin, J. Vickery, J. Jankowski, P. Villoslada, H. S. Markus, K. Wang, C. G. Mathew, J. Wason, C. N. A. Palmer, H.-E. Wichmann, R. Plomin, E. Willoughby, A. Rautanen, J. Winkelmann, M. Wittig, R. C. Trembath, J. Yaouanq, A. C. Viswanathan, H. Zhang, N. W. Wood, R. Zuvich, P. Deloukas, C. Langford, A. Duncanson, J. R. Oksenberg, M. A. Pericak-Vance, J. L. Haines, T. Olsson, J. Hillert, A. J. Ivinson, P. L. De Jager, L. Peltonen, G. J. Stewart, D. A. Hafler, S. L. Hauser, G. McVean, P. Donnelly, A. Compston, Genetic risk and a primary role for cell-mediated immune mechanisms in multiple sclerosis. *Nature* **476**, 214–*219* (2011).

3. T. Olsson, L. F. Barcellos, L. Alfredsson, Interactions between genetic, lifestyle and environmental risk factors for multiple sclerosis. *Nat. Rev. Neurol.* **13**, 25–36 (2017).

4. S. S. Soldan, P. M. Lieberman, Epstein-Barr virus and multiple sclerosis. *Nat. Rev. Microbiol.*, **21**, 51–64 (2022).

5. A. Langer-Gould, J. Wu, R. Lucas, J. Smith, E. Gonzales, L. Amezcua, S. Haraszti, L. H. Chen, H. Quach, J. A. James, L. F. Barcellos, A. H. Xiang, Epstein-Barr virus, cytomegalovirus, and multiple sclerosis susceptibility: A multiethnic study. *Neurology* **89**, 1330–1337 (2017).

6. K. Bjornevik, M. Cortese, B. C. Healy, J. Kuhle, M. J. Mina, Y. Leng, S. J. Elledge, D. W. Niebuhr, A. I. Scher, K. L. Munger, A. Ascherio, Longitudinal analysis reveals high prevalence of Epstein-Barr virus associated with multiple sclerosis. *Science* **375**, 296–301 (2022).

7. E. Sundqvist, P. Sundström, M. Lindén, A. K. Hedström, F. Aloisi, J. Hillert, I. Kockum, L. Alfredsson, T. Olsson, Epstein-Barr virus and multiple sclerosis: Interaction with HLA. *Genes Immun.* **13**, 14–20 (2012).

8. A. K. Hedström, J. Huang, N. Brenner, J. Butt, I. Kockum, T. Waterboer, T. Olsson, L. Alfredsson, Low sun exposure acts synergistically with high Epstein-Barr nuclear antigen 1 (EBNA-1) antibody levels in multiple sclerosis etiology. *Eur. J. Neurol.* **28**, 4146–4152 (2021).

9. A. K. Hedström, J. Huang, N. Brenner, J. Butt, J. Hillert, T. Waterboer, I. Kockum, T. Olsson, L. Alfredsson, Smoking and Epstein-Barr virus infection in multiple sclerosis development. *Sci. Rep.* **10**, 10960 (2020).

10. A. K. Hedström, J. Huang, A. Michel, J. Butt, N. Brenner, J. Hillert, T. Waterboer, I. Kockum, T. Olsson, L. Alfredsson, High levels of Epstein-Barr virus nuclear antigen-1-specific antibodies and infectious mononucleosis act both independently and synergistically to increase multiple sclerosis risk. *Front. Neurol.* **10**, 1368 (2020).

11. A. K. Hedström, I. Lima Bomfim, J. Hillert, T. Olsson, L. Alfredsson, Obesity interacts with infectious mononucleosis in risk of multiple sclerosis. *Eur. J. Neurol.* **22**, 578-e38 (2015).

12. International Multiple Sclerosis Genetics Consortium (IMSGC), A. H. Beecham, N. A. Patsopoulos, D. K. Xifara, M. F. Davis, A. Kemppinen, C. Cotsapas, T. S. Shah, C. Spencer, D. Booth, A. Goris, A. Oturai, J. Saarela, B. Fontaine, B. Hemmer, C. Martin, F. Zipp, S. D'Alfonso, F. Martinelli-Boneschi, B. Taylor, H. F. Harbo, I. Kockum, J. Hillert, T. Olsson, M. Ban, J. R. Oksenberg, R. Hintzen, L. F. Barcellos; Wellcome Trust Case Control Consortium (WTCCC); International IBD Genetics Consortium (IIBDGC), C. Agliardi, L. Alfredsson, M. Alizadeh, C. Anderson, R. Andrews, H. B. Søndergaard, A. Baker, G. Band, S. E. Baranzini, N. Barizzone, J. Barrett, C. Bellenguez, L. Bergamaschi, L. Bernardinelli, A. Berthele, V. Biberacher, T. M. C. Binder, H. Blackburn, I. L. Bomfim, P. Brambilla, S. Broadley, B. Brochet, L. Brundin, D. Buck, H. Butzkueven, S. J. Caillier, W. Camu, W. Carpentier, P. Cavalla, E. G. Celius, I. Coman, G. Comi, L. Corrado, L. Cosemans, I. Cournu-Rebeix, B. A. C. Cree, D. Cusi, V. Damotte, G. Defer, S. R. Delgado, P. Deloukas, A. di Sapio, A. T. Dilthey, P. Donnelly, B. Dubois, M. Duddy, S. Edkins, I. Elovaara, F. Esposito, N. Evangelou, B. Fiddes, J. Field, A. Franke, C. Freeman, I. Y. Frohlich, D. Galimberti, C. Gieger, P.-A. Gourraud, C. Graetz, A. Graham, V. Grummel, C. Guaschino, A. Hadjixenofontos, H. Hakonarson, C. Halfpenny, G. Hall, P. Hall, A. Hamsten, J. Harley, T. Harrower, C. Hawkins, G. Hellenthal, C. Hillier, J. Hobart, M. Hoshi, S. E. Hunt, M. Jagodic, I. Jelčić, A. Jochim, B. Kendall, A. Kermode, T. Kilpatrick, K. Koivisto, I. Konidari, T. Korn, H. Kronsbein, C. Langford, M. Larsson, M. Lathrop, C. Lebrun-Frenay, J. Lechner-Scott, M. H. Lee, M. A. Leone, V. Leppä, G. Liberatore, B. A. Lie, C. M. Lill, M. Lindén, J. Link, F. Luessi, J. Lycke, F. Macciardi, S. Männistö, C. P. Manrique, R. Martin, V. Martinelli, D. Mason, G. Mazibrada, C. M. Cabe, I.-L. Mero, J. Mescheriakova, L. Moutsianas, K.-M. Myhr, G. Nagels, R. Nicholas, P. Nilsson, F. Piehl, M. Pirinen, S. E. Price, H. Quach, M. Reunanen, W. Robberecht, N. P. Robertson, M. Rodegher, D. Rog, M. Salvetti, N. C. Schnetz-Boutaud, F. Sellebjerg, R. C. Selter, C. Schaefer, S. Shaunak, L. Shen, S. Shields, V. Siffrin, M. Slee, P. S. Sorensen, M. Sorosina, M. Sospedra, A. Spurkland, A. Strange, E. Sundqvist, V. Thijs, J. Thorpe, A. Ticca, P. Tienari,

C. van Duijn, E. M. Visser, S. Vucic, H. Westerlind, J. S. Wiley, A. Wilkins, J. F. Wilson, J. Winkelmann, J. Zajicek, E. Zindler, J. L. Haines, M. A. Pericak-Vance, A. J. Ivinson, G. Stewart, D. Hafler, S. L. Hauser, A. Compston, G. McVean, P. De Jager, S. J. Sawcer, J. L. McCauley, Analysis of immune-related loci identifies 48 new susceptibility variants for multiple sclerosis. *Nat. Genet.* **45**, 1353–1360 (2013).

13. T. Schneider-Hohendorf, L. A. Gerdes, B. Pignolet, R. Gittelman, P. Ostkamp, F. Rubelt, C. Raposo, B. Tackenberg, M. Riepenhausen, C. Janoschka, C. Wünsch, F. Bucciarelli, A. Flierl-Hecht, E. Beltrán, T. Kümpfel, K. Anslinger, C. C. Gross, H. Chapman, I. Kaplan, D. Brassat, H. Wekerle, M. Kerschensteiner, L. Klotz, J. D. Lünemann, R. Hohlfeld, R. Liblau, H. Wiendl, N. Schwab, Broader Epstein-Barr virus-specific T cell receptor repertoire in patients with multiple sclerosis. *J. Exp. Med.* **219**, e2022065010252022c (2022).

14. L. I. Levin, K. L. Munger, M. V. Rubertone, C. A. Peck, E. T. Lennette, D. Spiegelman, A. Ascherio, Multiple sclerosis and Epstein-Barr virus. *JAMA* **289**, 1533–1536 (2003).

15. P. Sundström, M. Nyström, K. Ruuth, E. Lundgren, Antibodies to specific EBNA-1 domains and HLA DRB1*1501 interact as risk factors for multiple sclerosis. *J. Neuroimmunol.* **215**, 102–107 (2009).

16. B. Ayoglu, N. Mitsios, I. Kockum, M. Khademi, A. Zandian, R. Sjoberg, B. Forsstrom, J. Bredenberg, I. Lima Bomfim, E. Holmgren, H. Gronlund, A. O. Guerreiro-Cacais, N. Abdelmagid, M. Uhlen, T. Waterboer, L. Alfredsson, J. Mulder, J. M. Schwenk, T. Olsson, P. Nilsson, Anoctamin 2 identified as an autoimmune target in multiple sclerosis. *Proc. Natl. Acad. Sci. U.S.A.* **113**, 2188–2193 (2016).

17. K. Tengvall, J. Huang, C. Hellstrom, P. Kammer, M. Bistrom, B. Ayoglu, I. Lima Bomfim, P. Stridh, J. Butt, N. Brenner, A. Michel, K. Lundberg, L. Padyukov, I. E. Lundberg, E. Svenungsson, I. Ernberg, S. Olafsson, A. T. Dilthey, J. Hillert, L. Alfredsson, P. Sundstrom, P. Nilsson, T. Waterboer, T. Olsson, I. Kockum, Molecular mimicry between Anoctamin 2 and Epstein-Barr virus nuclear antigen 1 associates with multiple sclerosis risk. *Proc. Natl. Acad. Sci. U.S.A.* **116**, 16955–16960 (2019).

18. T. V. Lanz, R. C. Brewer, P. P. Ho, J.-S. Moon, K. M. Jude, D. Fernandez, R. A. Fernandes, A. M. Gomez, G.-S. Nadj, C. M. Bartley, R. D. Schubert, I. A. Hawes, S. E. Vazquez, M. Iyer, J. B. Zuchero, B. Teegen, J. E. Dunn, C. B. Lock, L. B. Kipp, V. C. Cotham, B. M. Ueberheide, B. T. Aftab, M. S.

Anderson, J. L. DeRisi, M. R. Wilson, R. J. M. Bashford-Rogers, M. Platten, K. C. Garcia, L. Steinman, W. H. Robinson, Clonally expanded B cells in multiple sclerosis bind EBV EBNA1 and GlialCAM. *Nature* **603**, 321–327 (2022).

19. M. Hecker, B. Fitzner, M. Wendt, P. Lorenz, K. Flechtner, F. Steinbeck, I. Schröder, H.-J. Thiesen, U. K. Zettl, High-density peptide microarray analysis of IgG autoantibody reactivities in serum and cerebrospinal fluid of multiple sclerosis patients. *Mol. Cell. Proteomics* **15**, 1360–1380 (2016).

20. S. S. Ousman, B. H. Tomooka, J. M. van Noort, E. F. Wawrousek, K. C. O'Connor, D. A. Hafler, R. A. Sobel, W. H. Robinson, L. Steinman, Protective and therapeutic role for alphaB-crystallin in autoimmune demyelination. *Nature* **448**, 474–479 (2007).

21. J. M. van Noort, M. Bsibsi, W. H. Gerritsen, P. van der Valk, J. J. Bajramovic, L. Steinman, S. Amor, Alphab-crystallin is a target for adaptive immune responses and a trigger of innate responses in preactive multiple sclerosis lesions. *J. Neuropathol. Exp. Neurol.* **69**, 694–703 (2010).

22. M. Bsibsi, L. A. N. Peferoen, I. R. Holtman, P. J. Nacken, W. H. Gerritsen, M. E. Witte, J. van Horssen, B. J. L. Eggen, P. van der Valk, S. Amor, J. M. van Noort, Demyelination during multiple sclerosis is associated with combined activation of microglia/macrophages by IFN-γ and alpha B-crystallin. *Acta Neuropathol.* **128**, 215–229 (2014).

23. J. M. van Noort, A. C. van Sechel, J. J. Bajramovic, M. el Ouagmiri, C. H. Polman, H. Lassmann, R. Ravid, The small heat-shock protein alpha B-crystallin as candidate autoantigen in multiple sclerosis. *Nature* **375**, 798–801 (1995).

24. M. Bronge, K. A. Högelin, O. G. Thomas, S. Ruhrmann, C. Carvalho-Queiroz, O. B. Nilsson, A. Kaiser, M. Zeitelhofer, E. Holmgren, M. Linnerbauer, M. Z. Adzemovic, C. Hellström, I. Jelcic, H. Liu, P. Nilsson, J. Hillert, L. Brundin, K. Fink, I. Kockum, K. Tengvall, R. Martin, H. Tegel, T. Gräslund, F. Al Nimer, A. O. Guerreiro-Cacais, M. Khademi, G. Gafvelin, T. Olsson, H. Grönlund, Identification of four novel T cell autoantigens and personal autoreactive profiles in multiple sclerosis. *Sci. Adv.* **8**, eabn1823 (2022).

25. M. Lindskog, J. Rockberg, M. Uhlen, F. Sterky, Selection of protein epitopes for antibody production. *Biotechniques* **38**, 723–727 (2005).

26. R. Hohlfeld, K. Dornmair, E. Meinl, H. Wekerle, The search for the target antigens of multiple sclerosis, part 1: Autoreactive CD4+ T lymphocytes as pathogenic effectors and therapeutic targets. *Lancet Neurol.* **15**, 198–209 (2015).

27. M. Bronge, A. Kaiser, C. Carvalho-Queiroz, O. B. Nilsson, S. Ruhrmann, E. Holmgren, T. Olsson, G. Gafvelin, H. Grönlund, Sensitive detection of antigen-specific T-cells using bead-bound antigen for in vitro re-stimulation. *MethodsX* **6**, 1635–1641 (2019).

28. D. Lodygin, M. Hermann, N. Schweingruber, C. Flugel-Koch, T. Watanabe, C. Schlosser, A. Merlini, H. Korner, H.-F. Chang, H. J. Fischer, H. M. Reichardt, M. Zagrebelsky, B. Mollenhauer, S. Kugler, D. Fitzner, J. Frahm, C. Stadelmann, M. Haberl, F. Odoardi, A. Flugel, β-Synuclein-reactive T cells induce autoimmune CNS grey matter degeneration. *Nature* **566**, 503–508 (2019).

29. I. Jelcic, F. Al Nimer, J. Wang, V. Lentsch, R. Planas, A. Madjovski, S. Ruhrmann, W. Faigle, K. Frauenknecht, C. Pinilla, R. Santos, C. Hammer, Y. Ortiz, L. Opitz, H. Gronlund, G. Rogler, O. Boyman, R. Reynolds, A. Lutterotti, M. Khademi, T. Olsson, F. Piehl, M. Sospedra, R. Martin, Memory B cells activate brain-homing, autoreactive $CD4^+$ T cells in multiple sclerosis. *Cell* **175**, 85–100.e23 (2018).

30. R. Planas, R. Santos, P. Tomas-Ojer, C. Cruciani, A. Lutterotti, W. Faigle, N. Schaeren-Wiemers, C. Espejo, H. Eixarch, C. Pinilla, R. Martin, M. Sospedra, GDP-l-fucose synthase is a $CD4^+$ T cell-specific autoantigen in DRB3*02:02 patients with multiple sclerosis. *Sci. Transl. Med.* **10**, eaat4301 (2018).

31. M. Saraste, T.-L. Penttilä, L. Airas, Natalizumab treatment leads to an increase in circulating CXCR3-expressing B cells. *Neurol. Neuroimmunol. Neuroinflamm.* **3**, e292 (2016).

32. E. Kemanetzoglou, E. Andreadou, CNS demyelination with TNF-α blockers. *Curr. Neurol. Neurosci. Rep.* **17**, 36 (2017).

33. J. D. Lünemann, I. Jelcić, S. Roberts, A. Lutterotti, B. Tackenberg, R. Martin, C. Münz, EBNA1-specific T cells from patients with multiple sclerosis cross react with myelin antigens and co-produce IFN-gamma and IL-2. *J. Exp. Med.* **205**, 1763–1773 (2008).

34. J. Wang, I. Jelcic, L. Mühlenbruch, V. Haunerdinger, N. C. Toussaint, Y. Zhao, C. Cruciani, W. Faigle, R. Naghavian, M. Foege, T. M. C. Binder, T. Eiermann, L. Opitz, L. Fuentes-Font, R. Reynolds, W. W. Kwok, J. T. Nguyen, J.-H. Lee, A. Lutterotti, C. Münz, H.-G. Rammensee, M. Hauri-Hohl, M. Sospedra, S. Stevanovic, R. Martin, HLA-DR15 molecules jointly shape an autoreactive T cell repertoire in multiple sclerosis. *Cell* **183**, 1264–1281.e20 (2020).

35. K. W. Wucherpfennig, J. L. Strominger, Molecular mimicry in T cell-mediated autoimmunity: Viral peptides activate human T cell clones specific for myelin basic protein. *Cell* **80**, 695–705 (1995).

36. N. R. Jog, M. T. McClain, L. D. Heinlen, T. Gross, R. Towner, J. M. Guthridge, R. C. Axtell, G. Pardo, J. B. Harley, J. A. James, Epstein-Barr virus nuclear antigen 1 (EBNA-1) peptides recognized by adult multiple sclerosis patient sera induce neurologic symptoms in a murine model. *J. Autoimmun.* **106**, 102332 (2020).

37. R. Mechelli, J. Anderson, D. Vittori, G. Coarelli, V. Annibali, S. Cannoni, F. Aloisi, M. Salvetti, J. A. James, G. Ristori, Epstein-Barr virus nuclear antigen-1 B-cell epitopes in multiple sclerosis twins. *Mult. Scler.* **17**, 1290–1294 (2011).

38. M. T. McClain, E. C. Rapp, J. B. Harley, J. A. James, Infectious mononucleosis patients temporarily recognize a unique, cross-reactive epitope of Epstein-Barr virus nuclear antigen-1. *J. Med. Virol.* **70**, 253–257 (2003).

39. A. Pugliese, Autoreactive T cells in type 1 diabetes. *J. Clin. Invest.* **127**, 2881–2891 (2017).

40. A. Dawoodji, J.-L. Chen, D. Shepherd, F. Dalin, A. Tarlton, M. Alimohammadi, M. Penna-Martinez, G. Meyer, A. L. Mitchell, E. H. Gan, E. Bratland, S. Bensing, E. S. Husebye, S. H. Pearce, K. Badenhoop, O. Kämpe, V. Cerundolo, High frequency of cytolytic 21-hydroxylase-specific CD8+ T cells in autoimmune Addison’s disease patients. *J. Immunol.* **193**, 2118–2126 (2014).

41. A. Lanzavecchia, Antigen-specific interaction between T and B cells. *Nature* **314**, 537–539 (1985).

42. J. B. Rothbard, M. P. Kurnellas, S. Brownell, C. M. Adams, L. Su, R. C. Axtell, R. Chen, C. G. Fathman, W. H. Robinson, L. Steinman, Therapeutic effects of systemic administration of chaperone αB-crystallin associated with binding proinflammatory plasma proteins. *J. Biol. Chem.* **287**, 9708–9721 (2012).

43. A. Arac, S. E. Brownell, J. B. Rothbard, C. Chen, R. M. Ko, M. P. Pereira, G. W. Albers, L. Steinman, G. K. Steinberg, Systemic augmentation of alphaB-crystallin provides therapeutic benefit twelve hours post-stroke onset via immune modulation. *Proc. Natl. Acad. Sci. U.S.A.* **108**, 13287–13292 (2011).

44. J. B. Rothbard, X. Zhao, O. Sharpe, M. J. Strohman, M. Kurnellas, E. D. Mellins, W. H. Robinson, L. Steinman, Chaperone activity of α B-crystallin is responsible for its incorrect assignment as an autoantigen in multiple sclerosis. *J. Immunol.* **186**, 4263–4268 (2011).

45. J. Bhattacharyya, E. G. Padmanabha Udupa, J. Wang, K. K. Sharma, Mini-alphaB-crystallin: A functional element of alphaB-crystallin with chaperone-like activity. *Biochemistry* **45**, 3069–3076 (2006).

46. J. G. Ghosh, M. R. Estrada, J. I. Clark, Interactive domains for chaperone activity in the small heat shock protein, human alphaB crystallin. *Biochemistry* **44**, 14854–14869 (2005).

47. Natalizumab: AN 100226, anti-4alpha integrin monoclonal antibody. *Drugs R D* **5**, 102–107 (2004).

48. A. C. Karlsson, J. N. Martin, S. R. Younger, B. M. Bredt, L. Epling, R. Ronquillo, A. Varma, S. G. Deeks, J. M. McCune, D. F. Nixon, E. Sinclair, Comparison of the ELISPOT and cytokine flow cytometry assays for the enumeration of antigen-specific T cells. *J. Immunol. Methods* **283**, 141–153 (2003).

49. C. Wang, Y. K. Chou, C. M. Rich, J. M. Link, M. E. Afentoulis, J. M. van Noort, E. F. Wawrousek, H. Offner, A. A. Vandenbark, AlphaB-crystallin-reactive T cells from knockout mice are not encephalitogenic. *J. Neuroimmunol.* **176**, 51–62 (2006).

50. N. M. Thoua, J. M. van Noort, D. Baker, A. Bose, A. C. van Sechel, M. J. van Stipdonk, P. J. Travers, S. Amor, Encephalitogenic and immunogenic potential of the stress protein alphaB-crystallin in Biozzi ABH (H-2A(g7)) mice. *J. Neuroimmunol.* **104**, 47–57 (2000).

51. S. Ellmerich, M. Mycko, K. Takacs, H. Waldner, F. N. Wahid, R. J. Boyton, R. H. M. King, P. A. Smith, S. Amor, A. H. Herlihy, R. E. Hewitt, M. Jutton, D. A. Price, D. A. Hafler, V. K. Kuchroo, D. M. Altmann, High incidence of spontaneous disease in an HLA-DR15 and TCR transgenic multiple sclerosis model. *J. Immunol.* **174**, 1938–1946 (2005).

52. K. B. Abdul-Majid, J. Jirholt, C. Stadelmann, A. Stefferl, P. Kjellén, E. Wallström, R. Holmdahl, H. Lassmann, T. Olsson, R. A. Harris, Screening of several H-2 congenic mouse strains identified H-2(q) mice as highly susceptible to MOG-induced EAE with minimal adjuvant requirement. *J. Neuroimmunol.* **111**, 23–33 (2000).

53. R. Weissert, E. Wallström, M. K. Storch, A. Stefferl, J. Lorentzen, H. Lassmann, C. Linington, T. Olsson, MHC haplotype-dependent regulation of MOG-induced EAE in rats. *J. Clin. Invest.* **102**, 1265–1273 (1998).

54. C. Papeix, S. Vukusic, R. Casey, N. Debard, B. Stankoff, S. Mrejen, Z. Uhry, E. Van Ganse, A. Castot, M. Clanet, C. Lubetzki, C. Confavreux; TYSEDMUS and OFSEP Group, Risk of relapse after natalizumab withdrawal: Results from the French TYSEDMUS cohort. *Neurol. Neuroimmunol. Neuroinflamm.* **3**, e297 (2016).

55. L. Hosang, R. C. Canals, F. J. van der Flier, J. Hollensteiner, R. Daniel, A. Flügel, F. Odoardi, The lung microbiome regulates brain autoimmunity. *Nature* **603**, 138–144 (2022).

56. H. M. Long, J. Zuo, A. M. Leese, N. H. Gudgeon, H. Jia, G. S. Taylor, A. B. Rickinson, CD4+ T-cell clones recognizing human lymphoma-associated antigens: Generation by in vitro stimulation with autologous Epstein-Barr virus-transformed B cells. *Blood* **114**, 807–815 (2009).

57. A. C. van Sechel, J. J. Bajramovic, M. J. van Stipdonk, C. Persoon-Deen, S. B. Geutskens, J. M. van Noort, EBV-induced expression and HLA-DR-restricted presentation by human B cells of alpha B-crystallin, a candidate autoantigen in multiple sclerosis. *J. Immunol.* **162**, 129–135 (1999).

58. A. K. Hedström, M. Bäärnhielm, T. Olsson, L. Alfredsson, Tobacco smoking, but not Swedish snuff use, increases the risk of multiple sclerosis. *Neurology* **73**, 696–701 (2009).

59. C. Engler, R. Kandzia, S. Marillonnet, A one pot, one step, precision cloning method with high throughput capability. *PLOS ONE* **3**, e3647 (2008).

60. M. Bronge, S. Ruhrmann, C. Carvalho-Queiroz, O. B. Nilsson, A. Kaiser, E. Holmgren, C. Macrini, S. Winklmeier, E. Meinl, L. Brundin, M. Khademi, T. Olsson, G. Gafvelin, H. Grönlund, Myelin oligodendrocyte glycoprotein revisited-sensitive detection of MOG-specific T-cells in multiple sclerosis. *J. Autoimmun.* **102**, 38–49 (2019).

61. L. M. Topping, L. Romero-Castillo, V. Urbonaviciute, H. Bolinsson, F. I. Clanchy, R. Holmdahl, B. T. Bäckström, R. O. Williams, Standardization of antigen-emulsion preparations for the induction of autoimmune disease models. *Front. Immunol.* **13**, 892251 (2022).

62. M. Uhlen, L. Fagerberg, B. M. Hallstrom, C. Lindskog, P. Oksvold, A. Mardinoglu, A. Sivertsson, C. Kampf, E. Sjostedt, A. Asplund, I. Olsson, K. Edlund, E. Lundberg, S. Navani, C. A.-K. Szigyarto, J. Odeberg, D. Djureinovic, J. O. Takanen, S. Hober, T. Alm, P.-H. Edqvist, H. Berling, H. Tegel, J. Mulder, J. Rockberg, P. Nilsson, J. M. Schwenk, M. Hamsten, K. von Feilitzen, M. Forsberg, L. Persson, F. Johansson, M. Zwahlen, G. von Heijne, J. Nielsen, F. Ponten, Proteomics. Tissue-based map of the human proteome. *Science* **347**, 1260419 (2015).
